# Cometary dust: the diversity of primitive refractory grains

**DOI:** 10.1098/rsta.2016.0260

**Published:** 2017-05-29

**Authors:** D. H. Wooden, H. A. Ishii, M. E. Zolensky

**Affiliations:** 1 NASA Ames Research Center, Moffett Field, CA 94035-0001, USA; 2 University of Hawaii, Hawai’i Institute of Geophysics and Planetology, Honolulu, HI 96822, USA; 3 NASA Johnson Space Center, ARES, X12 2010 NASA Parkway, Houston, TX 77058-3607, USA

**Keywords:** comets, dust, interplanetary dust particles, UltraCarbonaceous Antarctic MicroMeteorites, *Stardust*, *Rosetta*, protoplanetary disc

## Abstract

Comet dust is primitive and shows significant diversity. Our knowledge of the properties of primitive cometary particles has expanded significantly through microscale investigations of cosmic dust samples (anhydrous interplanetary dust particles (IDPs), chondritic porous (CP) IDPs and UltraCarbonaceous Antarctic micrometeorites, *Stardust* and *Rosetta*), as well as through remote sensing (*Spitzer* IR spectroscopy). Comet dust are aggregate particles of materials unequilibrated at submicrometre scales. We discuss the properties and processes experienced by primitive matter in comets. Primitive particles exhibit a diverse range of: structure and typology; distribution of constituents; concentration and form of carbonaceous and refractory organic matter; Mg- and Fe-contents of the silicate minerals; sulfides; existence/abundance of type II chondrule fragments; high-temperature calcium–aluminium inclusions and ameboid-olivine aggregates; and rarely occurring Mg-carbonates and magnetite, whose explanation requires aqueous alteration on parent bodies. The properties of refractory materials imply there were disc processes that resulted in different comets having particular selections of primitive materials. The diversity of primitive particles has implications for the diversity of materials in the protoplanetary disc present at the time and in the region where the comets formed.

This article is part of the themed issue ‘Cometary science after Rosetta’.

## 1. Conspectus: changing paradigms

**Figure 1.**
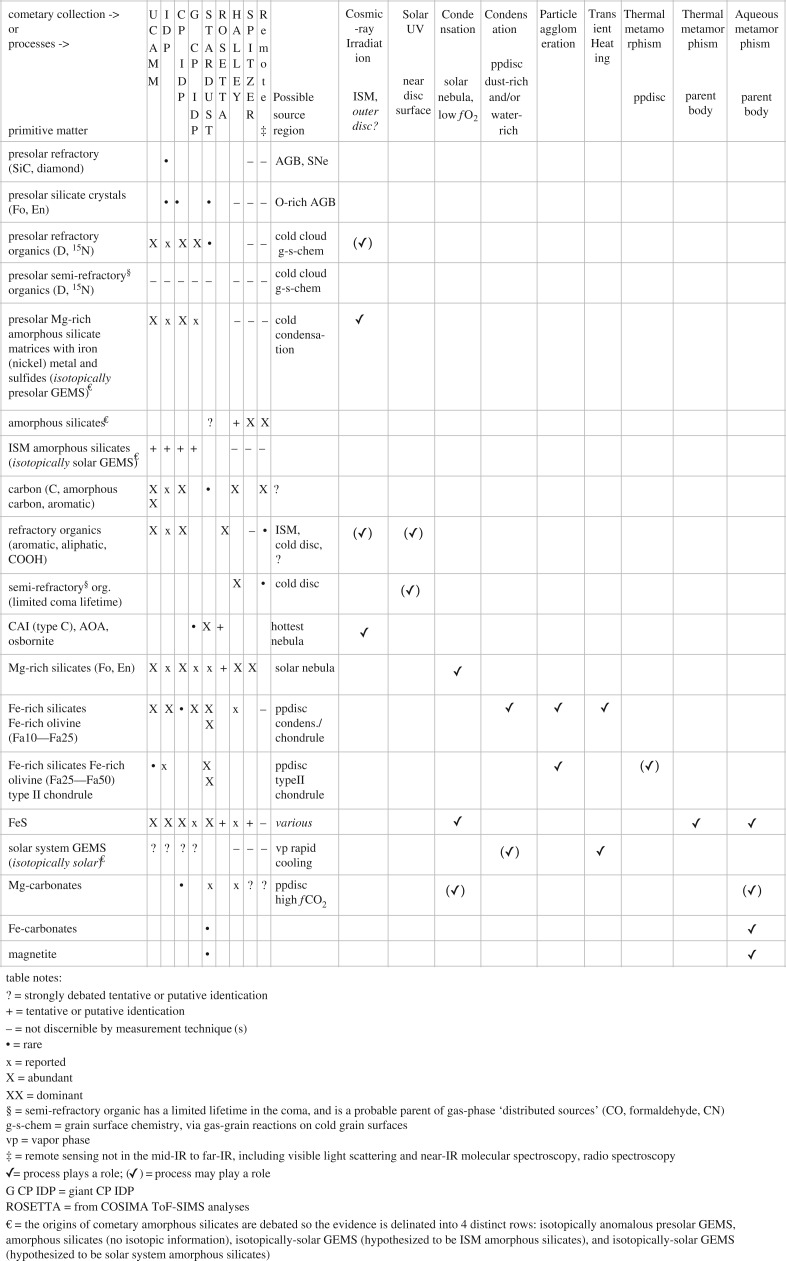
Table: diversity of primitive matter in cometary dust.

The astrophysical connections to comet dust and the clues that comet dust provide for comet origins and for planet-forming processes have evolved considerably over the last 12 years. The diversity of primitive materials in comets has stimulated cross-disciplinary investigations among cometary, chondritic and extraterrestrial materials such as interplanetary dust particles (IDPs) and Antarctic micrometeorites (AMMs) [1–4]. We define primitive cometary materials as unequilibrated, aqueously unaltered and minimally thermally altered (in the nebula). We discuss the diversity of primitive matter in comets. We choose to adopt the path presented by Libourel *et al.* [5], wherein they describe, ‘we define primitive matter in our Solar System through a parameterization scheme based on the amount and intensity of processes the matter underwent since its delivery to or its formation in the Solar System instead of defining it on the basis of its age only. Of course at some point, the time of formation comes into play, but first, the matter considered the most primitive in terms of a specific process should show little evidence for subsequent modification by other processes, etc. The most primitive is not necessarily the oldest, as usually considered, but the least affected in the number or kind of processes it underwent inside the limit of our Solar System’. We adopt this framework in our discussion of the primitive materials in cometary refractory dust. In discussing different aspects of refractory dust, we highlight aspects that help to indicate how and where cometary primitive materials likely formed or the regimes from which they probably were transported. The regimes from which the materials were transported were the sites where processes occurred over either long times (cold regimes) or short times (shocks) or possibly near-steady state such as in the early solar nebula where Mg-rich crystals condensed. Figure 1 is a tabularized description of cometary primitive materials and the processes for which there is evidence that they likely experienced. Also in figure 1, we attempt to indicate in what cometary reservoirs the primitive materials have been identified, which is an ever-evolving subject as more micro- and nano-scale laboratory investigations are reported for IDPs and *Stardust* samples and as *Rosetta* discoveries are reported.

To provide a conceptual framework, we distinguish between different regions of the protoplanetary disc environs: pre-solar (includes molecular cloud stage), outer nebular (after the formation of the proto-sun but still cold enough to harbour the supervolatiles CO and CH_4_), the near-surface and disc atmosphere (where UV radiation can penetrate) [6], as well as the inner nebular (hot, condensation), and the chondrule-forming region that we think of as extending to larger radii than the ‘inner disc’ (a region of rapid, high-temperature excursions and shocks). We do not assign these regions timescales, but we note that condensation of the highest temperature refractory grains (calcium–aluminium inclusions (CAIs) and ameboid-olivine aggregates (AOAs), §4) occurred over a limited duration of about 1.5 Myr [7], early chondrule formation was co-temporal with CAIs and chondrule formation extended to *ca* 4.5 Myr [8]. There is overlap between pre-solar materials [9] and early nebula materials time-wise since pre-solar materials were still accreting into the outer disc while accretion occurred onto the proto-sun. A weak shock in the outer disc persisted probably through 10^5^ yr or through the disc eras defined by FU Orionis outbursts due to density change between the infalling cloud and the outer disc. In order to be incorporated into cometary materials, presolar materials had to survive passage through this infalling cloud–outer disc interface [10].

A large part of the reflections are based on results from the NASA *Stardust Mission* and the ESA *Rosetta Mission*, as well as results on extraterrestrial samples (IDPs, CP IDPs and UltraCarbonaceous Antarctic micrometeorites (UCAMMs)). Each collection and/or analyses method biases the results and it is important to keep this in mind in order to not misinterpret or over interpret some results. *Stardust* samples of cometary dust from the coma of comet 81P/Wild 2 (81P) were collected in aerogel, during the flyby at around 6 km s^−1^, and were returned to the Earth [11]. Indeed, the velocity of the particles ‘slamming’ into aerogel biased the collection: the bulbous tracks are from exploded porous particles, submicrometre grains are altered extensively, the identification of glassy phases is highly questionable, and most all the carbonaceous content is at least partially altered or is lost. Large (5–30 μm) *Stardust* grains survived intact [12], and are at track termini and called terminal grains and comprise 65% of all aerogel tracks. A few *Stardust* organic particles piggy-backed behind terminal grains during aerogel capture and survived to be studied in detail [13]. *Stardust* terminal grains include type II chondrule fragments [14] and indicate the incorporation of late-formation disc materials and therefore imply, for some comets, late comet formation in our protoplanetary disc (ppdisc) [15].

For *Rosetta* results and mainly COSIMA analyses, the main bias that must be taken into account is the large size of the mass spectrometer time-of-flight secondary ion mass spectrometer (ToF-SIMS) beam, which has a diameter of around 40 μm. The COSIMA beam is comparable in diameter to the biggest *Stardust* terminal particles. Given the spatial scale of mineralogical diversity observed, for example, in IDPs and in UCAMMs on submicrometre scales, it is almost impossible for COSIMA to identify single minerals, and it may be almost blind to minor phases or minerals in each analysed particle. Furthermore, the high Fe-content of grain assemblages measured in the COSMIA beam may be attributable to FeS rather than high Fe-content silicates [16]. Thus, for *Stardust* and *Rosetta* (and theoretically, for any analysis), not seeing something does not necessarily mean that it does not exist.

The structure of this review article is to lead with a *conspectus*, a comprehensive view. We describe the paradigms of the past [17], present and projected future—the evolution of information and thoughts about refractory matter in cometary dust and its primitive nature. The topic is rich with information. The results span a large range of specialties and are not easy to summarize without losing the complexity inherent to the subjects. Thus, our choice is to lead with the conspectus, an abridged version of the whole story, and provide the sections that follow as the electronic supplementary material supporting the ideas explained in the conspectus. The electronic supplementary material provides sections on chondrule types, chondrule-matrix complementarity and the depletion pattern relevant to thinking about the potential connections between cometary and asteroidal materials. Our aim is to better engage investigators of various disciplines in the rich dialogue about the properties of cometary refractory particles because they provide key data and insights into the properties, formation and evolution of dust from the interstellar medium (ISM) and from our protoplanetary disc.

### (a) Old paradigm

Comet dust was considered to be mostly fine-grained, that is having a preponderance of submicrometre- to micrometre-sized components of discrete single-mineral grains or grains aggregated into micrometre-sized and larger porous particles. Studies of cometary dust via the *in situ* Halley flybys, IR spectroscopy of cometary comae, and laboratory examinations of cometary anhydrous IDPs and of cometary anhydrous CP IDPs [17–20] provided similar frameworks for contemplating comet dust as collections of materials inherited from the ISM and condensates from the early solar nebula. Inherited materials included Mg–Fe amorphous silicates^1^ as well as amorphous carbon (§1a(v)) and organics, whereas condensates were Mg-rich crystalline silicates. Crystalline silicates in comets heralded the importance of radial mixing in the ppdisc of hot inner disc material to the cold outer regimes (10–30 AU) [25–28] where cometary nuclei accreted refractory dust grains along with volatile and supervolatile ices [29,30].

#### (i) Mg-rich crystalline silicates

Mg-rich crystals were our best analogues for condensates from the early ‘solar nebula’, i.e. from early in our ppdisc evolution when high mass accretion rates evaporated all dust and strong turbulent mixing produced a gas reservoir of solar composition [17,20,31]. Mg-rich crystalline silicates, dominantly forsterite and occasionally enstatite, have distinct resonances that were detected in comets and in external protoplanetary discs, e.g. comet C/1995 O1 (Hale–Bopp) [32,33] compared with HD 100546 [34,35]. The comet–disc connection and the implications for disc radial transport were amplified by the discovery of external systems with inner discs enriched in crystals compared with their outer discs [36]. Mg-rich crystalline silicates were created prior to later chondrule-forming events, which introduced geochemical complications into the reservoir of refractory grains that included Fe-rich crystalline silicates. That is, the old paradigm did not genuinely consider chondrules as cometary dust constituents and did not highlight Fe-rich crystalline silicates as cometary primitive dust.

Mg-rich crystalline silicates also were considered to possibly/probably form by annealing of amorphous silicates. The annealing scenario was favoured by ppdisc modellers [26] because as time progresses from ∼10^5^ to 10^6^ yr, the volume of the disc hot enough to condense crystals dramatically shrinks as the mass accretion rate declines and the mid-plane temperatures fall [37]. Increasing the mass of condensed Mg-rich crystals becomes harder with time as does radial transport by diffusion out to the comet-forming zone [25,26]. As the ppdisc evolves, radial transport transitions to mechanisms governed by turbulence and aerodynamics and moving crystals out to the comet-forming regimes becomes even more challenging [27,28,38]. At larger distances beyond the condensation front for Mg-rich crystalline silicates, shocks can occur and drive temperatures to more than 900–1200 K where amorphous silicates anneal to crystals [39], possibly preferably annealing to forsterite [40]. Thus, shock-heating increases the volume of the ppdisc that can make crystals via annealing [41]. One challenge to the annealing scenario, however, was that crystalline silicates were Mg-rich but their presumed ISM precursors were Mg–Fe amorphous silicates, which likely would anneal to Mg–Fe crystals [42]. Annealing under conditions of low oxygen fugacity (log( *f*_O_2__), defined in §2) can reduce Fe in the mineral/material to nFe particles [43]. The annealing scenario, however, might work for small, ≲1  μm size, Mg–Fe amorphous silicate precursors if the grains were heated in the ‘dry and low dust enriched’ pre-shock gas for about an hour prior to the rapid spike in shock temperature, such that Fe could be reduced and diffuse to the surface prior the annealing event that did the crystallization [44]. Amorphous silicates are readily annealed to crystals; changing their compositions in the process of annealing requires these conjectured special circumstances.

The paucity of crystalline silicates in the ISM, i.e. less than or equal to 2.5% [45,46] or less than or equal to 5% [47], substantiated the idea that crystals formed in the ppdisc and amorphous silicates were inherited from the ISM. Along lines-of-sight through the ISM, absorption bands can be fitted by spherical Mg–Fe amorphous silicates^2^ [45–47] and non-spherical Mg amorphous silicates [50]. ISM gas-phase depletion studies also show both Mg-silicates and Fe–Mg-silicates [17,51,52]. O-rich AGB stars readily form and contribute Mg–Fe amorphous silicates and Mg-crystalline silicates to the ISM [53], but the near-absence of crystals in the ISM justifies an amorphizing mechanism. An important point is that the lifetimes of grains in the ISM are too long compared with the efficiency of shock sputtering and destruction so grains must re-condense in cold, dense molecular clouds [54,55]. Recent experiments show the viability of this ‘ISM cold condensation’ scenario [52,56,57]. Thus, the dominant component of ISM dust condensed in molecular clouds, was released, reprocessed in the ISM by shocks and cosmic rays, and recycled into and out of molecular clouds [58]. Cosmic-ray exposure causes amorphization [48] and transforms Fe in the crystal lattice to nanophase Fe^0^ (nFe) [17,44,48,52] and is hypothesized to produce nFeS [59]. In fact, amorphous olivine or amorphous pyroxene are very unstable and would rapidly crystallize. For an amorphous silicate to be metastable, it needs to be off-stoichiometry, which also usually means highly ‘defected’ and having lots of dangling bonds, such as occurs by cosmic-ray damage [48,52,60]. Cosmic-ray exposure typically is thought to occur in the ISM. It is speculative to suggest that solar cosmic-rays associated with X-ray flares from the young Sun could amorphize grains in the tenuous atmosphere of the ppdisc, or in jets/outflows [61]; alternatively, X-ray flares may be possible energy source for annealing silicates in the near-surface disc layers [62]. The general view is that crystallization is efficient and that amorphous silicates have not been heated to the point of annealing, which implies amorphous silicates have been preserved since their amorphization or since their formation as an amorphous material.^3^

#### (ii) GEMS, the amorphous silicates in cometary refractory dust

One important amorphous silicate component of anhydrous IDPs is the GEMS, Glass with Embedded Metal and Sulfides [18]. Two competing theories about GEMS origins are reviewed: their formation in the ISM and their formation in our ppdisc.

Many GEMS show tracks from radiation exposure [18]. Some GEMS have non-solar isotopes that clearly label them as ‘presolar’ amorphous silicates [63–65], inherited from the ISM or ISM–prenatal cold molecular cloud core. GEMS-rich regions of anhydrous IDPs can have anomalous compositions from cosmic-ray exposure and show a range of non-solar oxygen isotopic ratios [66]. GEMS are our best analogue for inherited ISM amorphous silicates [2,67,68].^4^ GEMS have spectral signatures that are comparable to ISM amorphous silicates [22,23,70]. GEMS with measurable presolar isotopic ratios are identical in all other aspects to the rest of the GEMS population, so ‘GEMS remain the best candidate for surviving interstellar amorphous silicates’ [23].

Heating of GEMS from IDPs to 900°C creates Fe-rich olivine (crystals) [71]. GEMS are easily destroyed by aqueous or thermal alteration, which is prevalent on asteroidal parent bodies. Presolar signatures^5^ for organics are seen as enrichments in D/H, ^15^N/^14^N [73] or anomalous O-isotopic ratios [73] (also see §4). Presolar signatures are destroyed most easily by thermal alteration (GR Huss 2015, personal communication) and also are destroyed by aqueous alteration. During Earth-atmospheric entry of IDPs, the heating of GEMS causes sulfides to melt and migrate to their surfaces, and Mg and S become depleted [75–77]. The sulfur content of GEMS grains provides clues to their origins, but there are complications since S is modified by atmospheric entry (see also §6).

CP IDPs that appear to have suffered the least atmospheric heating are the best cometary samples in which to study the GEMS [78]. When GEMS and presolar materials are found together they signify preservation of inherited materials. There is an interesting correlation: the CP IDPs that have a lot of GEMS also have a lot of presolar grains (some of them GEMS) [73]. GEMS-rich IDPs are called ‘ultra-primitive’ cometary CP IDPs, which include CP IDPs purportedly collected from the dust stream of comet 26P/Grigg–Skjellerup (26P) [65,72]. The highly GEMS-rich IDPs have Mg-rich crystalline silicates [72,73,79]. Mg-rich crystals in GEMS-rich IDPs are typically enstatite (Mg-rich pyroxene) with some forsterite (Mg-rich olivine) [79]. The properties of comae dust deduced from thermal models of cometary IR spectra have strong similarities to the properties of CP IDPs (§8), but with forsterite dominating rather than enstatite [80]. A main point is cometary primitive matter contains ISM and ppdisc materials [17,20].

On the other hand, the formation of amorphous silicates in the ppdisc is the competing hypothesis for GEMS origins [81]. The formation of amorphous silicates in the ppdisc via condensation or via shocks, however, has severe challenges. As described by Abreu & Brearley [82], experiments show that amorphous silicates form by vapour-phase nucleation in timescales of microseconds [83] but that these glasses would anneal to crystals unless temperatures dropped rapidly to below 700–1050 K. Shock models predict gases and dust do not cool quickly enough to account for GEMS-formation. This is because once a particle passes through the shock front and subsequently slows down to the post-shock gas velocity, gas and solids become thermally coupled and cool in lockstep only as fast as they can leave the shock front, which means cooling rates are approximately 100 K h^−1^ [82,84]. Note that glass (mesostasis) is abundant in chondritic materials, but it is an igneous by-product (§2).

The disc-origins theory for GEMS arises from studies of their elemental and isotopic abundances. The results of [81,85] based on the element-to-Si ratios are (i) GEMS have element-to-Si ratios too low to match ISM grain compositions and (ii) GEMS show an order of magnitude variations in elemental abundances. Origins for GEMS in the ppdisc is argued for as follows: the observed variations in GEMS’ elemental abundances are too large for a common origin in the ISM, via chemical and isotopic *extensive/complete homogenization*, and most GEMS’ O-isotopes are near meteoritic values (near solar) and meteorites formed in the solar system. Thus, GEMS formed in our ppdisc [85]: the ‘majority of GEMS grains have chemical, mineralogical, and isotopic properties that are inconsistent with … the average properties inferred for interstellar silicate grains’. The conclusion is that GEMS formed in our ppdisc [81,85]. Up to 6% (4/239 GEMS studied) are *bona fide* presolar GEMS based on their O-isotopic anomalies [85]. Whether the lack of isotopic anomalies is sufficient evidence for non-ISM origins is debated, with opponents calling for ISM cold condensation (re-formation, §1(ii)). We note that only about 1% of crystalline silicates have O-isotopic presolar signatures (§4) so the GEMS grains population has a greater relative abundance of presolar signatures compared to crystalline silicates. There is significant contention, however, over the use of element-to-Si ratios for samples collected and stored in silicone oil, which is known to be an uncontrollable contaminant [86–88].^6^ The newest data for GEMS grains are based on dry collection on polyurethane sheets without silicone oil [91], and the element-to-Si ratios are consistent with the studies of GEMS collected in silicone oil [81]. However, only two GEMS grains from dry collection have been analysed, so the statistics remain far too limited for a definitive assessment of the effects of removing silicone oil from the collection process, as the authors also indicate [91]. Dry collections of IDPs in the stratosphere, unfortunately, are in a hiatus because of funding challenges, so it will be a while until additional dry collections are obtained.

The interpretation of the degree of diversity is fuelling the controversy of GEMS origins. There is controversy over whether elemental abundances are being completely homogenized by ISM processing/re-formation. The wide compositional ranges of individual GEMS that have been shown to be presolar by isotope anomalies refutes the argument that elemental abundances should be *completely homogenized* in the ISM. Destruction of dust, much of it down to the atom/ion level, in the ISM means that isotope anomalies are mixed/diluted towards the mean. The recycling of matter through molecular clouds [58], and possibly through ppdiscs [61], shreds isotopic anomalies and produces isotopically solar dust. We favour the hypothesis that condensation/re-formation in dense clouds still occurs in the context of chemical affinities such that reformed solids need not be completely homogeneous elementally. This supports arguments that even GEMS that lack isotopic anomalies (not possessing presolar O-isotopic signatures) are surviving amorphous ISM silicates [59,68]. Reports on the CM2 Paris chondrite describe GEMS-like materials in the most primitive portions of the matrix adjacent to regions where GEMS-signatures are obliterated by parent body alteration [92]. Identifying ISM amorphous silicates in cometary materials is much more probable than identifying ISM amorphous silicates in chondrites (meteorites) because of how *prevalently GEMS-like grains are destroyed.*

Another reason to believe that GEMS formed by radiation exposure is that we see similar materials in one chondritic meteorite, Ningqiang, for which high-temperature formation is impossible. The GEMS-like material in Ningqiang probably is from the ppdisc and not from the ISM [93,94].

#### (iii) Primary minerals: abundances and Mg-, Fe-contents

A census by Zolensky & Barrett [95] of primary minerals in 15 anhydrous IDPs shows that the major minerals are olivine, pyroxene and FeS [95], and that olivine and pyroxene in anhydrous IDPs contain a range of Fe-contents from Mg-rich to ‘chondritic’ (Mg=Fe): specifically, anhydrous IDPs have pyroxene and olivine compositions in the ranges of En100–En46 and of Fo100–Fo50 (equivalently, Fa0–Fa50) [95].^7^ This means the olivine spans from Mg-rich to Fe-rich, where meteoriticists consider Fe-rich as Fe/(Mg+Fe)
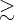
0.1 and chondritic to be Mg≈Fe.

**Figure 2.**
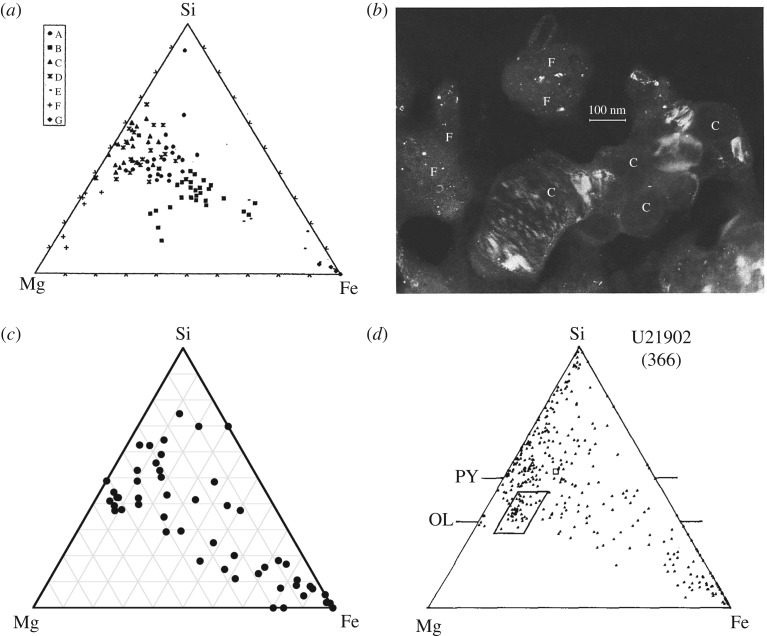
Ternary diagrams. Comparison of grain compositions from 1P/Halley (PUMA-1 on *Vega*), CP IDP U2-20GCA and IDP U219 C2. IDP U219 C2 was a point-count analyses (footnote 6). (*a*) The elemental composition of cometary particles (from 1P/Halley) in the system Mg–Si–Fe. Different symbols mark members of different groups that were obtained by cluster analysis (adapted from [98], fig. 7). (*c*) Atom fraction ternary of submicrometre grains (femtorocks) from U2-20GCA, a cluster IDP. The large heterogeneity at the submicrometre scale is remarkable and relatively unconstrained by mineralogical stoichiometry. The large dispersion is similar to data on particle compositions obtained at comet Halley (reproduced with permission from [99], fig. 1. Copyright © 2004 D. E. Brownlee). (*b*) Darkfield electron micrograph of a region of (anhydrous IDP) U219C2 containing both coarse- (labelled C) and fine- (labelled F) grained components. (*d*) Mg–Si–Fe ternary plot for U219C2. The cluster of data points (boxed area) from an assemblage of coarse-grained crystals like those (labelled C) shown in (*b*) (reproduced with permission from [79], fig. 6. Copyright © 1989 Elsevier).

In contrast to Zolensky & Barrett, other authors emphasized Mg-rich crystals in anhydrous IDPs with ∼Fo100–Fo90 and ∼En100–En90 [19,33,79,87,96,97]. Bradley *et al.* [79] did point count analyses over entire IDP sections^8^ with high concentrations of points falling on En100–En90 and Fo100–Fo90 in ternary diagrams for the anhydrous fine-grained IDPs with high GEMS contents (not recognized as GEMS at that point in time). The spot size for those measurements was 20 nm. Figure 2 shows ternary diagrams where each point is an individual grain analysis for comet 1P/Halley (Halley) in (figure 2*a*) and for a CP IDP in (figure 2*c*). For comparison, figure 2*b*,*d* shows the ternary diagram of an anhydrous IDP where the *rectangle* indicates a high concentration of points that are for Fo90 coarse-grained crystals. Point-count analyses provide multiple analyses of the same crystal and represents a volume sampling (figure 2*b*,*d*) [79]. Multiple analyses per crystal could have over-populated their Fe–Mg–Si ternary diagrams or the frequency distribution of Fe–Mg-contents for the crystals relative to figure 2*a*. In comparison, the analyses by Zolensky & Barrett [95] permitted only one analysis per crystal, thereby presenting an analysis of the sample by number and not by volume (nor by wt%). A similar oversampling occurred in early in analyses of *Stardust* (SD) samples, where the same terminal forsterite grains were multiply measured by different investigators having slices of the same track. Thus, the frequency distribution of Fe-contents of *Stardust* olivine lost the peak at Fo100 between [100,101] and more recent work [12].

Bradley *et al.* reveal higher Fe-content crystals in anhydrous coarser-grained IDPs with lower GEMS contents. [79]. In Halley dust, as reviewed by Rietmeijer *et al.* [102], assemblages were interpreted as being dominated by Mg-rich silicates with less amounts of Fe(Ni)-sulfides and very few FeO grains [17,103]. However, if Halley’s smallest grains are divided into ‘heavy’ and ‘light’ (more than 10^−13^ g and less than 5×10^−16^ g) then the heavy particles had Fe/(Fe+Mg) ≈ 0.4 or Fa40 [104]. Fa40 is in the range of compositions discussed by Zolensky & Barrett for olivine grains in their sample of 15 anhydrous IDPs. More than 200 IDPs were examined (by point-count analyses) and ternary diagrams were generated for a combination of smooth (typically hydrous) and porous (typically anhydrous) IDPs [87]. For just the anhydrous coarse-grained IDPs in their sample [80], the Fe–Mg–Si ternary diagram motivates their description and interpretation of the larger Mg-rich crystals versus the smaller Mg–Fe crystals, ‘The high abundance of Mg silicates suggest a high temperature origin for the coarse-grained fraction of IDPs and possibly a different origin for the submicrometre material that more closely matches chondritic elemental composition. Among the most interesting properties of the large mineral grains are their minor element abundances, the composition of their inclusions and the nature of particles bonded to their surfaces’. As above, we note GEMS-rich CP IDPs have Mg-rich crystals of enstatite and forsterite [73,79]. We agree that anhydrous IDPs deserve a second look now, especially to make measurements of minor elements in olivine and pyroxene.

Studies from two decades ago described CP IDPs as having porosities of approximately 40% and having subgrains sized from 0.005 to 0.5 μm [105–107].

#### (iv) Carbonaceous matter, refractory organics

Studies of CP IDPs include intriguing reports of aliphatic-carbon-dominated rims on siliceous subgrains [108]. Also, rims on silicates, sulfides and carbonates are carbonaceous matter with aromatic bonds and C=O functional groups, which cannot have formed by Fischer–Tropsch-like reactions and which probably formed by irradiation of ice coatings [109], similar to organic-formation pathways suggested by Ciesla & Sandford [6]. The rims are speculated to be the ‘glue’ that holds aggregates together [108].

When large D/H ratios are found in CP IDPs, they are in domains of aliphatic and aromatic carbon, and aliphatic hydrocarbons are observed in far greater abundance in the D-rich compared with the D-poor regions; variations in D/H may be due to loss of very D-rich volatile phases during atmospheric entry [110]. Anhydrous IDPs contain refractory organic matter [111] with some similarities with and some distinctions with the organics observed along lines-of-sight through the ISM [112,113]. Distinctions between *Stardust* and ISM organics may indicate *Stardust* organics formed in the outer ppdisc [11]. Organic vesicles (hollow spheres) in *Stardust* samples and larger cosmic spherules with presolar isotopic signatures (^16^O-rich) also are probably cometary materials. We denote this cometary organic matter as refractory, as it has survived since release from the coma as well as its traverse through the interplanetary medium. There are semi-volatile organics^9^ associated with dust grains that have limited lifetimes in cometary comae [44,114,115] and such species contribute to ‘distributed sources’ of comae molecules, such as CO, HNC, CN and formaldehyde (H_2_CO) [116]; cf. [44]. CP IDPs have regions of presolar grains and predominantly are considered cometary materials, although atmospheric entry may modify some labile organic materials. The first detection of organic matter on the surface of a comet was made by the *Rosetta* VIRTIS near-IR spectrometer for comet 67P/Churyumov–Gerasimenko (67P) [117–119] and among the compositions suggested for the broad 3.2 μm feature is R–COOH or carboxylic acid. Carboxylic acid also is seen in anhydrous IDPs [111].

#### (v) Amorphous carbon and hydrogenated amorphous carbon

Carbon XANES (X-ray absorption near-edge spectroscopy) of anhydrous IDPs reveals *various forms of the element of carbon* including amorphous carbon, highly disordered carbon (poorly graphitized carbon) and large domains of aromatic carbon bonds with no or few peripheral H-bonds [120]. Comet Halley had carbonaceous-only, siliceous-only and mixed composition dust. Of the carbonaceous-only dust in Halley, 25% was elemental carbon [121] but its structure was not decipherable by mass spectrometry. ‘Amorphous carbon’ and all the other macromolecular carbonaceous matter found in IDPs, UCAMMs, chondrites and in 67P dust, are part of the so-called ‘refractory organics’. The macromolecular matter that only contains C and H is in the ‘amorphous carbon’ category. The low levels of H in ’amorphous carbon’ make it distinct from ‘hydrogenated amorphous carbon’ (HAC), which has higher levels of H and discernible 3.2–3.6 μm features from peripheral bonds. The macromolecular matter outside of the ‘amorphous carbon’ category also contains different amounts of heteroatoms (O, N, S, etc.) for the natural materials found in extraterrestrial particles.

Amorphous carbon has no crystalline structure but has short-range order and medium-range order where medium-range order is particularly important in amorphous carbon [122]. Amorphous carbon [122] and HAC are distinguishable by the size of their aromatic islands, which may be linked by aliphatic structures, including sp, sp^2^ and sp^3^ carbon atoms [123]. Many techniques cannot distinguish organic from inorganic amorphous carbon. Owing to the processing of the dust grains in the ISM, the composition and structure is constantly modified; large aromatic domains are involved in these processes both as precursors of condensation and products of grain disintegration [123]. Asymptotic Giant Branch (AB) carbon stars condense C-rich dust and post-AGB stars have emission features from carbon dust with aromatic bonds (3.28 μm) and from aliphatic bonds (3.4 μm) [124]. The 3.28 μm band can be attributed to PAHs or HACs (e.g. [125]). In astrophysical contexts, HACs typically mean very small aromatic units (two to eight rings, [123,126]). Another formation mechanism for the 3.4 μm band is long-term space-FUV photo-processing of organic residues ([127], e.g. EURECA samples), where organic residues result from warmed-up laboratory-irradiated ices. An important point is that the strong (20% contrast with the continuum) broad 3.4 μm band in EURECA samples is not seen in cometary near-IR spectra.

Spectral absorption features for aromatic C–H bonds (3.28 μm) are distinguishable in a few IDPs but the aliphatic bonds (approx. 3.4 μm) are commonly detected [111,113,128]. The lack of an aromatic band could be explained if the carbonaceous matter is poorly graphitized carbon or very large PAH molecules with few peripheral H bonds [128]. The organic matter in IDPs with a higher proportion of aliphatic bonds relative to low molecular weight PAHs is distinctly different from primitive meteorites that have aromatic and aliphatic bonds [128]. Transmission spectra of whole IDPs (10 μm sized) show the 10 μm silicate bands but lack the 3.4 μm band; but when they are crushed or thin-sectioned they do show the 3.4 μm absorption band, suggesting that the aliphatic bond-carrier is destroyed on the external periphery of the particles perhaps by UV radiation [108]. Transmission spectra of nine thin-sectioned IDPs show 50 nm thick aliphatic-bond-dominated rims on the subgrains (components) of aggregate particles [129,130]. In summary, spectral absorption features near 3.4 μm are well measured for IDPs [113] where they are distinctly attributable to −CH_2_ and −CH_3_ aliphatic bonds [108,111,113,128], but they have yet to be identified unequivocally in cometary near-IR spectra.

The existence of cometary spectral features in the 3.2–3.6 μm range from the *thermal emission* from PAHs and/or HACs, and from carbonaceous matter with −CH2 or −*CH*3 bonds was hypothesized in the 1990s [131]. Later, the 3.4 μm band was called the ‘cometary organic feature’ [17,132]. The *EPOXI* Mission flyby of 103P/Hartley 2 reported organics associated with the CO_2_-rich part of the coma compared to the water-dominated part of the coma [133], fig. 6 as well as in the *Deep Impact*-induced coma of comet 9P/Tempel 1 [132,134]. There are strong gas-phase molecular emission lines in this wavelength region, however, that only are spectrally resolved at high resolution (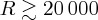). In the high-resolution data, the dust continuum is reported but there are no reports of broad features from solid-state organics [135]. At such high spectral resolution, broad solid-state emission features might be weak compared with the high-contrast molecular emission lines. Thus, the identification of emission features from solid-state organics in cometary comae [136] is controversial.

Amorphous carbon is used to fit the ubiquitously present warm featureless pseudo-continuum emission in the near-IR spectra of comets, i.e. featureless thermal emission in the opacity gap for silicates at wavelengths shorter than of 7.5 μm [17,41]. Amorphous carbon is a material that potentially is inherited from the ISM and its origin via organic or inorganic (e.g. AGB-star condensation followed by ISM sputtering and re-formation) pathways is indecipherable by most laboratory methods.

#### (vi) Carbon abundance

**Figure 3.**
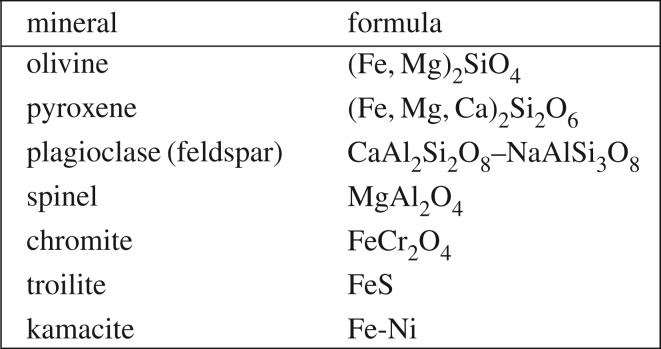
Formulae for common minerals in chondrules [200], table 1.

A series of reports in 1992–1994 by Thomas *et al.* discussed the abundance of carbon in anhydrous IDPs. In 19 anhydrous IDPs, carbon abundances were reported to be 5–23 wt%, which was consistent with [87,137], and they noted ‘the high carbon abundance in some anhydrous IDPs is seemingly incompatible with an origin from known chondritic materials’ [138]. Of the 19 anhydrous IDPs, 11 were thin sectioned and the abundance of carbon was found to be correlated with the dominant silicate mineral [139], fig. 3: olivine-dominated have carbon around 2–12 wt% (≃CI), olivine-pyroxene have around 7–17 wt%, pyroxene-dominated have 10–25 wt% carbon (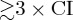). Furthermore, the ‘high carbon fragments appear to be very fine-grained, whereas the low carbon regions are rather large, individual grains with little fine-grain material’ [139], which also was found true for multiple fragments of individual cluster IDPs [140–142]. A range from 1 wt% –25 wt% carbon corresponds to 40–50 vol%, with one carbon-rich particle having 47 wt% carbon and up to 90 vol% carbon [141]. Considering multiple groups’ reports, the average C/Si ratio for anhydrous IDPs was around 2×CI [139].

We draw focus in this review to refractory organic matter in comets (including ‘carbon’) because the high abundance of carbon distinguishes cometary dust from asteroidal dust where carbonaceous chondrites may have up to 5% carbon (electronic supplementary material, section (a)). Amorphous carbon is destroyed or transformed in the ppdisc and cannot be formed in the inner ppdisc because the oxygen fugacity is too high, so amorphous carbon is considered part of the outer disc reservoir probably inherited from the ISM. In the ISM, carbon is depleted from the gas phase into dust [51], graphitic carbon contributes to the ISM extinction curve [47] and cosmic-ray bombardment can convert graphitic carbon to amorphous carbon [143]. Carbon is a ‘reducing agent’ whereby during a heating event C bonds with O and forms CO or CO_2_. The loss of O then can drive Mg–Fe silicates to more Mg-rich compositions; chondrule compositions may be driven by available carbon (electronic supplementary material, section (a), §5). Hence, the relative abundance of carbon in different cometary dust reservoirs may contribute to distinguishing formation mechanisms for the crystalline silicates. Cometary particles with Mg-rich crystalline silicates (probable condensates) may have more carbon than cometary particles with Fe-rich crystalline silicates (probable type II chondrule fragments). Fe-rich, type II chondrule olivine is a focus in the ‘new paradigm’ §1b. Lastly, grains with compositions like amorphous carbon would be optically highly absorbing so amorphous carbon has been the most referenced candidate material to account for the near-IR warm featureless ‘continuum’ emission that is ubiquitous in cometary comae, radiating in the near-IR opacity gap of silicates at shorter wavelengths than the so-called 10 and 20 μm bands [17,144].

*Stardust* samples have organic vesicles, large domains of aromatic carbon, and PAHs with about 20 C atoms [120]. *Stardust* has some domains of organic-dominated matter [13], better preserved when located behind a track terminal particle that passed through the aerogel without rotating, protecting it from the heat of capture which is severest at the entry site into the aerogel collecting medium. However, taking into account the destructive nature of the aerogel capture process at approximately 6 km s^−1^ and the analyses from the *Stardust* aluminium foil (strips holding the aerogel and also acting as collecting media), *Stardust samples do not have the high abundance of carbon that is witnessed in other cometary dust samples* [11].

The rather simple scenario of comets being combinations of amorphous carbon and Mg–Fe amorphous silicates, which are inherited from the ISM, and Mg-rich silicate crystals, which are early solar nebula condensates, is supported by thermal models of cometary IR spectra (§8) and by laboratory spectra of anhydrous IDPs [145]. IR spectral features in cometary comae are best modelled by submicrometre solid grains or by micrometre size and larger porous aggregates of submicrometre subgrains. Five compositions suffice to well-fit comet IR spectra: amorphous Mg–Fe silicates (amorphous olivine-like and amorphous pyroxene-like compositions), amorphous carbon and Mg-rich crystalline silicates [17,146–149]. The IR crystalline silicate resonances are best matched with forsterite and ortho-enstatite [32,150]. Laboratory spectra show that for increasing FeO contents the wavelengths of the spectral peaks shift to longer wavelengths [151–153] and these longer wavelengths do not match comet IR spectra [154,155].

Excellent spectral fitting requires varying crystal mass fractions for forsterite, except for one epoch of Hale–Bopp that displayed strong distinct spectral features of ortho-enstatite as well as forsterite [150]. Anhydrous CP IDPs with Mg-rich crystals are good analogues to modelled comet particles [17,20,146,154,156]. Modelled comet particles require moderate porosities of 65–80% [41,157], with increasing porosities for larger particles [41]. Aggregates of much higher porosity do not fit the observed *Spitzer* spectra of comets because at the highest porosities, temperatures of larger grains are too high since highly porous aggregates take on the temperatures of their monomers [158] and because the contrast in their spectral features do not fade with larger aggregate particle sizes [159]; larger cooler aggregate particles with weaker spectral features are needed in the particle size distributions in order to best-fit spectral energy distributions with wide spectral coverage (like *Spitzer*). Cometary silicate crystal mass fractions have typical values of 20–70% but range approximately from 0% for C/2006 P1 (McNaught) [160,161] to 79% for comet Hale–Bopp at perihelion [41,162] (see §8).

### (b) New paradigm

*Stardust* (SD) samples from comet 81P/Wild 2 (81P) significantly shifted the way we think about comet dust, because the majority of the mass collected and studied were relatively large (5–30  μm) [12,163] mineral grains or mineral assemblages. By number of track types in *Stardust* aerogel collecting media, there were more single large particles than aggregates as 65% were ‘carrot-shaped tracks’ from solid terminal particles more than 10 μm size and 35% were ‘bulbous tracks’ from porous aggregate particles. Some are 20 μm single mineral grains that are gigantic versions of the same mineral grains seen in CP IDPs and deduced for cometary comae from IR spectra. A 20 μm *Stardust* crystal can be around 1000× the volume of a fine-grained crystal such that (i) realistically, their sheer volume implies that they are not annealed assemblages of fine-grained (0.1–0.5 μm size) crystals and mixed silicates (GEMS, or radiation-damaged amorphous silicates) and (ii) many *Stardust* mineral grains are FeO-rich olivines.

FeO-rich olivines are unequilibrated with respect to the (Mg-rich) forsterite and enstatite, meaning (i) they did not form from the same nebular gases, and (ii) they did not exchange Fe–Mg atoms, so that (iii) they were not metamorphosed significantly by heat or by water (not aqueously altered) and formed in different ppdisc conditions. To date, no hydrated silicates (no phyllosilicates) have been identified in *Stardust* samples [14,101,164].^10^

Rare phases in *Stardust* that need explanation by aqueous alteration include some sulfides [167], magnetite [168] and Mg-carbonates [164,169]. The common belief is these rare grains formed by aqueously alteration that occurred on a different parent body from which these grains were collisionally ejected. Subsequent transportation to the comet-forming region allowed them to be incorporated into comet 81P. As discussed by Flynn *et al.* [170], small (≲0.02 to approx. 0.2 μm) Mg-carbonates were detected in *Stardust* [169,171] and in comet Halley [172], as well as in some anhydrous IDPs [129]. More area of *Stardust* picokeystones was searched (0.03 mm^2^) using C-XANES and no additional Mg-carbonates were detected [173]. The lack of phyllosilicates, which are signposts of aqueous alteration, motivates considering potential nebular sources for Mg-carbonates. Mg-carbonate condensation is potentially viable for specific conditions in the ppdisc that correspond to either enhanced CO_2_ at lower pressures and temperatures (*T*_gas_<450 K and *P*<10^−7^ bar) [174], such as above the mid-plane and in the 2–4 AU region [17]. Alternatively, experiments demonstrate Mg-carbonates can condense in CO_2_–H_2_O-rich vapour [175]. *The lack of hydrated silicates in* Stardust *and cometary CP IDPs is commensurate with our definition of primitive cometary particles as particles lacking aqueous alteration and being unequilibrated, i.e. assemblages of disparate oxygen fugacity minerals so as to be unequilibrated at submicrometre scales.*

The census of *Stardust* terminal grains has evolved since 2008–2011 as more studies are completed. Joswiak *et al.* [176] reported on *Stardust* large terminal grains [176]. Forsterite and enstatite, which probably are nebular condensates, are relatively common. A large amount of *Stardust* grains are CAI fragments and Fe-rich olivine (Fa30–Fa70), which cannot be considered condensates. In *Stardust* samples, refractory-rich *Stardust* assemblages such as the particle ‘Inti’ are similar to type C CAIs, AOAs (§4) and Al-rich chondrules in chondrites [177]. These assemblages, however, lack the most refractory type-A CAIs found in chondrites, so *Stardust* investigators suggest ‘comets may have preferentially accreted second generation refractory materials that had been moderately processed in the nebula’ [177]. Large numbers of *Stardust* terminal grains are Fe-rich olivine: Frank *et al.* [12] show a histogram of fayalite contents for the 5–30 μm-sized olivine grains and the histogram appears bimodal (figure 7). A similar analyses of different *Stardust* olivine grains populates the frequency distribution of the Fa0–Fa20 region [178]. Combining the two studies produces a fairly uniform (flat) frequency distribution for Fe-contents spanning Fa0 to Fa50 [179] (D Brownlee 2016, personal communication).

Within *Stardust* and chondrite Fe-rich olivine grains, moderately volatile elements have a wide-range of abundances with respect to solar composition (the composition of the Sun and the presumed composition of an early, fully mixed ppdisc). There is a particular focus on manganese (Mn). The FeO-contents and the MnO-contents of *Stardust* olivine are most similar to olivine in type II chondrules. The observed *Stardust* chondrule fragments are comparable to the size of microchondrules, i.e. to the lower size range of chondrite chondrules.^11^ Chondrules are millimetre size spherical-ish ‘balls of dust’ that were heated to near liquidus or in some cases heated all the way to liquidus. Chondrules constitute about 50% of the meteoritic record derived from asteroidal parent bodies.^12^ A giant CP IDP was observed to contain a similar wide range of olivine FeO- and MnO- contents as *Stardust* olivine [179,181]. Comparison of the in-depth studies of these two reservoirs of cometary dust promotes the idea that comets have a wide diversity of materials as well as the relatively small ‘body-to-body diversity’ between cometary dust reservoirs [179].

The FeO-contents and range of minor elements, in particular, the presence of moderately volatile elements including Mn, Ca, Cr, and the Fe–Mn relation in the olivines, are consistent with the olivine grains in primitive chondrules and matrix. The olivine grains in matrix are considered type II chondrule fragments. The similarities between the minor element abundances between *Stardust* olivines and the chondrule olivines from many chondrite classes suggest *Stardust* olivines are radially transported from reservoirs more diverse than what contributed to any single chondrite [182]. The studies of olivines in *Stardust* and in type II chondrules establishes a clear bridge between comet dust and asteroidal dust, and pushes the nature of the examinations and discussions of comet dust into the (more complicated) realm of geochemistry.^13^

### (c) Emerging paradigm

In contrast to the strong focus on comet–chondrite connections, recent measurements of giant CP IDPs and from *Rosetta* are reinvigorating the conversation about cometary materials as early ppdisc condensates (crystals as condensates versus crystals as melts), and as aggregate particles. The giant CP IDP (U2-20GCA), which is one giant CP IDP with a wide range of Mn-contents [181,183] report one ^16^O-rich enstatite crystal (1 μm × 2 μm) such as expected from an early ppdisc condensate: δ^17^O=−40±9‰, δ^18^O=−44±4‰. This giant CP IDP has a greater proportion of inner solar system ^16^O-rich phases compared with presolar grains in this CP IDP and to presolar grains in *Stardust* samples [183]. In this giant CP IDP, δ^15^N values range from 0 to 1500‰. Greater than 200 O-rich subgrains fall within the range of solar system materials, similar to *Stardust* samples [184].

The majority of *Rosetta* COSIMA and MIDAS studies of dust sampled from the coma of 67P, including COSIMA imaging [185] and compositional measurements of particles [16,186,187] and MIDAS atomic force microscope (AFM) imaging of particles and subgrains [188], are providing support to the view that cometary comae particles can be dominated by hierarchical aggregates of refractory materials. MIDAS and COSIMA, however, are not seeing the particles at the same scale. COSIMA sees the particles that are bigger than 10 μm, whereas MIDAS sees at smaller scales of less than 1 μm to up to 10 μm. At the COSIMA imager COSISCOPE’s spatial resolution (14 μm), the individual ‘grains’ are not distinguished [185]. The COSIMA team discusses the typology of the particles as compact aggregates (approx. 15%) and as disrupted aggregates (approx. 85%). For COSIMA, most particles appear to be disrupted aggregates of millimetre size. The typology of disrupted aggregates are designated ‘clusters’ with subclasses of ‘rubble piles’, ‘shattered clusters’, and ‘glued clusters’, and ‘compact’; their shapes on the collecting plates are reminiscent of pancakes, coral-reefs, flour-piles and stacked blocks with some gaps [185], respectively. The structures of particles are unresolved ‘micro-breccias’ [185]. Langevin *et al.* [185] compare *Rosetta* particles collected by COSIMA to descriptions of UCAMMs and ‘cosmic dust’ with porosities of more than 20% [11].

The particles (1–10 μm) analysed by MIDAS are made of smaller units (grains) that are more or less compacted to form ‘particles’. The particle ‘E’ reported [188] is in the ‘COSIMA size range’ and is big compared with A, B, C and D. This one special large particle studied by MIDAS has extremely high porosity and low fractal dimension [188]. Under 5 μm, rather compact subunits can be distinguished by MIDAS. The term ‘compact’ used by the COSIMA team does not have exactly the same meaning as for MIDAS and it applies for a few particles (all bigger than 10 μm) that most probably are compacted aggregates. MIDAS findings would tend to support that idea. In the literature, similar structures are called compact porous aggregates [148] as opposed to highly porous aggregates [159] like MIDAS’ particle ‘E’ [188].

The *Rosetta* COSMIA and MIDAS teams are describing the particles in the coma of 67P as *hierarchical aggregates*. Most are compact porous aggregates and aggregates at each size scale going down to 0.05 μm. *Rosetta*’s particles have porous structures like CP IDPs, which have crystalline minerals and aggregate-subcomponents that may be themselves multi-component ‘femtorocks’ of silicates and organics with and without core-mantle structure [99].

In stark contrast to *Rosetta*, most of *Stardust* samples were 5–30 μm solid mineral grains or fused mineral assemblages (rocks), found in the bottom of ‘carrot’-shape tracks. Only 35% of *Stardust* tracks were ‘bulbous’-shape tracks (or ‘turnip’-shape) from porous aggregates, with ‘bulbous’ being both large with long stylus tips and small without stylus [11,163,189]. Note Joswiak *et al.* [189] report distinctions between the mineralogy of grains in the two shapes of bulbous tracks compared with the carrot tracks. Studies of UCAMMs (§6) contribute to the ‘emerging paradigm’: there are important distinctions between *Stardust* and other cometary dust samples. *Even given the aerogel collection bias, we think the ¡textit¿Stardust* collection appears to have less elemental carbon and organics than other cometary samples.¡/textit¿^14^ Recall the Thomas *et al.* studies of anhydrous IDPs (§1a(v)) that showed a correlation between lower carbon content and less fine-grained material. Along these lines, *Stardust* has significantly less fine-grained material, which account for 35% of *Stardust* aerogel tracks, compared with the IDPs from the 26P/G-S stream that are dominated by fine-grained dominated. We speculate that if *Stardust* is dominated by coarse-grained dust (type II chondrule fragments [14]) and has less carbonaceous matter, then this correlates with primitive meteorites that have coarse-grained chondrule-fragments and less carbonaceous matter than some cometary samples. *Stardust* materials likely formed late in disc evolution, after greater than or equal to 2.6 Myr (‘Pixie’, §2). Some matter in UCAMMs and some GEMS-rich IDPs have ^16^O-enrichments indicative of presolar or early disc materials. In comparison, these signatures are significantly less abundant in *Stardust* samples.

The *Rosetta* COSIMA instrument identifies in the coma of 67P a component of the dust that is high molecular weight organic matter [190]. Two 100 μm size regions, ‘Kennith’ and ‘Juliette’ are reported on and are representative of seven other particles. COSIMA’s mass spectra of the organic matter in Kennith and Juliette are best represented by reference spectra of insoluble organic matter (IOM) in carbonaceous chondrites (Orgueil and Murchison); three aspects in which they are similar are that they both have high molecular weights, lack carbon-bearing ions with *m*/*z* ratios more than 50, and have lower H/C ratios. The COSIMA results call out the lack of lower molecular weight organics with higher H/C ratios including carboxylic acids, aliphatics, PAHs or amino acids [190]. This is in contrast to the frequent detection of carboxylic acids and aliphatic carbon in IDPs (e.g.[111]). The organic matter in 67P is distinct from carbonaceous chondrite (CC) IOM by its higher CH_*x*_^+^/C^+^ ratio, which translates to a higher H/C ratio.^15^ A higher H/C ratio is attributed to the comet being a more primitive body than CCs, because parent body processing tends to lower the H/C ratio in IOM in CCs [191]. Fray *et al.* suggest the source regions for comet 67P’s organic matter could be the ISM [192] or the cold outer ppdisc [6]. The only comparison based on experimental data that has been reported by Fray *et al.* is the one made with the IOM extracted from carbonaceous meteorites.

An interesting aspect of the diversity in IOM in cometary samples is the range of N/C ratios. The *Rosetta* COSIMA measurements cite the detection in the negative-ion spectra of peaks of the fragments CN^−^ and CNO^−^ but the C/N ratio is not given. *Stardust* samples have N/C ranging from 0.03 to up to 0.2–0.3 [13,193]. Meteoritic IOM have N/C ranging from 0.01 to 0.05 with 0.03 being typical [191]. Most IDPs and AMMs are in this range [194]. By contrast, a well-studied UCAMMs (DC65) has an N/C ratio of 0.12, which is a factor of more than four times greater than *Stardust* IOM. Such high N content in organic matter suggests its formation via energetic processing in a N-dominated environment. Dartois *et al*. hypothesize its origin on a N_2_-rich surface requiring the gravity of a large parent body in the Kuiper Belt or a small parent body in the extreme cold of the Oort cloud [195]. Either of these scenarios implies this level of N-enhancement in IOM should be rare compared with typical IOM that may form in the significantly greater volumes of the ISM or the outer disc.

In the coma of comet 67P, the *Rosetta* ROSINA mass spectrometer measured a wondrously diverse ‘zoo’ of gas-phase carbonaceous molecules including S-bearing molecules (the zoo’s skunks) [196]. The simplest molecules detected by ROSINA (H_2_S, OCS, SO, SO_2_ and C*S*_2_) are present in cometary ices [196–198], so there is not necessarily a genetic link to the S present in the dust. Amino acids and their precursors identified by ROSINA probably were made in ice coatings on dust grains [199]. We speculate that a bit more complex organic S-bearing molecules that are in the mass range of ROSINA could be more interesting than the simple molecules listed above and serve as a perspective for the comparison with organic sulfides or sulfur oxides possibly present in the solid-state organics in UCAMMs (to be verified, not given in the above list) (§6) or in the organic matter of carbonaceous chondrites.

**Figure 4.**
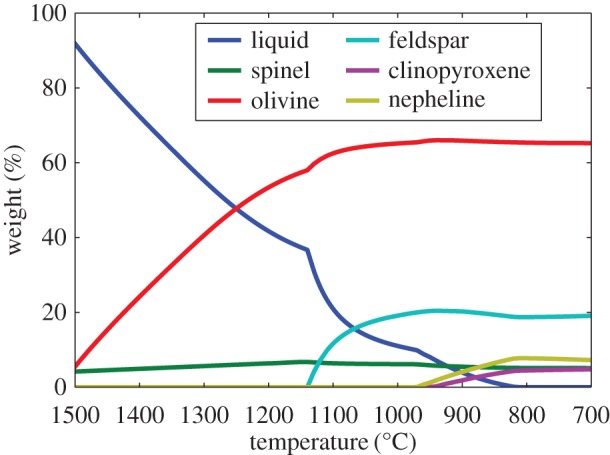
Iris crystallization sequence. MELTS (model) predicted phase abundances as a function of temperature for the fugacity log(log(*f*_O_2__)) = IW – 0.25, Na_2_O=3.5 wt%. At high temperature, olivine and spinel were co-forming (reproduced with permission from [14], fig. 19. Copyright © 2015 John Wiley & Sons, Inc.). (Online version in colour.)

Current and future reports of *in situ* measurements of the dust in the coma of comet 67P by *Rosetta* instruments will contribute to understanding the similarities and differences between comet dust reservoirs. *Rosetta*’s COSMIA shows 67P’s dust composition has a higher Fe/Mg ratio than the Fe/Mg ratio in CI chondrites [16], fig. 4. possibly because of a greater abundance of FeS compared with other cometary samples [16]. Only one CAI-like grain has been found [187]. Silicates appear to have high Fe-contents in COSIMA mass spectra but this may be a consequence of silicates being near co-spatial with abundant FeS [187]. 67P’s refractory organics have high-molecular-weight, as measured by *Rosetta*’s COSIMA, and are most similar to IOM in primitive chondrites and to refractory organic matter in UCAMMs (§6).

Overarching questions are: How typical are *Stardust* materials in other comets? That is, do all comets contain chondrule fragments and have such strong connections to chondrite matrix materials (type II chondrule fragments) as *Stardust*? Did all comets form as late as 81P? If comets formed late, then why/how did some comet reservoirs not become as populated in the products of chondrule-formation and instead have populations of presolar and early disc condensation?

## 2. Igneous particles: *Stardust* chondrule ‘Iris’

Gainsforth *et al.* summarize [14], ‘A compilation of grains observed in 16 aerogel tracks suggests that comet Wild∼2 contains a greater population of chondrule objects, especially type II chondrule objects, than other igneous refractory assemblages [189]’. We review three *Stardust* type II chondrule fragments, ‘Iris’, ‘Callie’ and ‘Torajiro’. One aim is to establish a context for comet observers who may not be as versed in geochemistry and meteoritics as those experts studying *Stardust* and extraterrestrial materials (IDPs, UCAMMs).

We provide some background terminology here. Minerals found in chondrules are itemized in figure 3. The olivine mineral group spans Mg-rich forsterite (Fo) (Mg_2_SiO_4_) to Fe-rich fayalite (Fa) (Fe_2_SiO_4_), which also are labelled, respectively, as Fo100 and Fo0 or equivalently Fa0 to Fa100. The mol% of forsterite is *X*_Fo_=Mg/(Mg + Fe) or alternatively the mol% of fayalite is *X*_Fa_=Fe/(Mg+Fe) such that *X*_*Fo*_+*X*_Fa_=100 mol%.^16^ Fe in olivine and pyroxene must be in FeO (as divalent Fe^2+^), so the term FeO-content is interchangeable with the term Fe-content.^17^ (FeO is wüstite.) IW denotes the iron-wüstite buffer above which FeO-bearing olivine forms. MgO-rich olivine forms at well below IW, and a log oxygen fugacity. A low oxygen fugacity, log( *f*_O_2__) = (IW − 3), is expected for the first condensation products in a gas of solar composition in §7. The notations and basic metrics that distinguish chondrules and chondrite classes are in §1a.

The well-studied 20 μm size *Stardust* type II chondrule ‘Iris’ (C2052,12,74) [14,15,201] is an igneous particle because it was heated to near liquidus and cooled slowly enough for minerals to crystallize in an equilibrium sequence. Fe-rich olivine contains Cr-spinel inclusions, and feldspar surrounds both olivine and spinel. The largest olivine is 7 × 11 μm and the FeO-content is uniform throughout such that there is no zoning (no gradient in Fe-content towards the edges), at the limit of the measurements Δ*X*_Fa_≤2–3. The crystallization geochemistry was modelled by Gainsforth *et al.* [14] using the MELTS code and assuming thermal equilibrium, which is reasonable given the lack of zoning. The porphyritic olivine (PO) texture indicates crystal growth from multiple nucleation sites and heating to near-liquidus temperature. By contrast, fully melted droplets have no remnant nucleation sites and have non-porphyritic textures. The MELTS model cooling sequence that best fits Iris is shown in figure 4. The crystallization sequence probably began near 1500°C (1773 K) with Cr-spinel (chromite, FeCr_2_O_4_), followed by spinel and FeO-rich olivine coevolution that reached near-equilibrium above 1000°C. Surrounding the olivine and spinel, high-Ca pyroxene ((Mg,Ca)SiO_3_) as well as Na-rich plagioclase (Na-rich end of the feldspar mineral group NaAlSi_3_O_8_—CaAlSi_3_O_8_ or albite—anorthite) formed and stayed in equilibrium from 1000°C to 900°C. Lower than 900°C, the melt fell out of equilibrium as evidenced by no more crystallization but instead the formation of glass or mesostasis (amorphous feldspar or amorphous plagioclase). Thus, Iris was quenched around 700–800°C. The amorphous phase was non-stoichiometric with similar composition as the crystalline albitic feldspar except for excess SiO_2_.

The Fe-content of the olivine began near Fa25 and increased during crystallization to Fa40 [14], fig. 21, due to the high oxygen fugacity of log(log(*f*_O_2__))=−13.3 (IW − 0.25 at 1000°C), at a pressure of 1 bar.^18^ The cooling rate was less than or equal to 100°C h^−1^ because higher cooling rates would have produced some zoning [14], fig. 22 and no zoning was measured in Iris. The final Fe-content of the olivine resulted from the available FeO in the melt. The moderately volatile element Mn was incorporated during crystallization and the MnO-content of the olivine also traced the bulk composition of the melt precursor materials. Iris has an olivine composition of Fa35–Fa40, with MnO=0.63 wt%, Cr_2_O_3_=0.16 wt% and CaO=0.3 wt%.

The olivine Fe-content categories Iris as a type II chondrule or type II chondrule fragment. The oxygen fugacity being close to IW can be explained by Iris forming in a high dust/gas environment where evaporated pre-existing silicate dust contributes oxygen to the gas phase. The MELTS results show that the bulk sodium content of Iris started with Na_2_O=3.5 wt% and 40% of the Na was lost during cooling from the melt in an open system behaviour for Na. Na is volatile and the fact that only 40% of the Na was lost means that the ambient partial pressure of Na was near equilibrium, which is commensurate with a dust enrichment of approximately 10^4^ [14,202].

A second *Stardust* chondrule ‘Callie’ consists mainly of olivine (Fa36–Fa41) and Callie also contains Na-rich feldspar (plagioclase) [14]. A third *Stardust* chondrule ‘Torajiro’ [203] consists mainly of olivine (Fa19–Fa20), low-Ca pyroxene (En86Wo3), small kamacite blebs (Fe, Ni-metal found in meteorites), and glass enriched in SiO_2_ and Al_2_O_3_ [14]. Compared with Iris, Torajiro formed at slightly higher temperatures and higher oxygen fugacity (1090°C, based on the olivine/spinel geothermometer, and log(log( *f*_O_2__))=−12(IW+1.4) [14]. Torajiro’s olivine is *X*_Fa_=10 mol%, which is lower than Iris at *X*_Fa_=36 mol%, even though Torajiro crystallized from a melt at higher oxygen fugacity. If we consider Iris and Torajiro as examples of type II chondrule formation, the oxygen fugacity of crystallization does not translate directly to Fe-content of the olivine but instead to the olivine-spinel system. This is in contrast to condensation sequences of solar nebula gases where olivine *X*_Fa_ mol% can be predicted from the oxygen fugacity in a gas of solar composition (§7) [26,204]. The outcome of the igneous systems depends on the cooling rate, composition of the precursor dust and the oxygen fugacity.

In Iris, the Ca-rich clino-pyroxene crystallized at less than or equal to 950°C and this pyroxene has Na- and Ca-enrichments and is slightly less enriched in Al and Ti. Iris’ Ca-rich pyroxene and olivine has similarities to Kool particles [14], as discussed in §3. Another *Stardust* chondrule, terminal grain 5 of C2061,1,113,5 has a Na-rich, Si-poor material that is similar to mesostatis in Al-diopside-rich chondrule fragments and is not linked to *Stardust* Kool particles [205]. Iris and Callie are distinguished from Kool particles by their larger sized crystals (‘coarse-grained’ texture) [14].

Bringing to mind the newest data, *Rosetta* COSIMA’s collecting plates reveal Na-enhancements that are characteristic of the locations of cometary particles [186]. Na probably is not associated with a silicate component because Na is not co-located with Si [16]. Thus, the lack of Si rules out Kool particles as *Rosetta*’s Na-rich material since Kool particles have olivine and pyroxene. One wonders about how the Na-rich, Si-poor phase in *Rosetta* would compare with the Na-rich, Si-poor material in *Stardust* chondrule C2061,1,113,5 [205].

A census of *Stardust* materials was made using STXM and Fe-XANES.^19^ Some Mg- and Fe-bearing siliceous matter was located on a trailing edge of Iris and analogies were drawn with GEMS [206]. Signatures in STXM mapping indicate similar material in the bottom of the tracks [207], and it was suggested that 50% of the mass of *Stardust* track matter is in this nearly invisible glassy phase [206]. However, we do not take the Stodolna *et al.* result [206] as definitive evidence for GEMS in *Stardust* because our research on aerogel capture indicates GEMS-like compositions, which are on average solar, are impact-produced debris from the mixing of aerogel, silicate and sulfide from larger particles [78]. The fundamental problem is that the *Stardust* capture conditions were exactly wrong for finding *bona fide* GEMS.^20^ In contrast to the fine-grained material in question, STXM and Fe-XANES shows *Stardust* has a higher Fe composition compared with CP IDPs because comet 81P is rich in iron sulfides (e.g. FeS) [209].

Nanoscale IR spectra from 8.7 to 12.5 μm (800–1150 cm^−1^) were obtained for Iris [201]. Spectral resonances were identified for regions of Iris characterized by ‘forsterite’-rich, anorthite-rich, albite-rich and albitic-glass materials [201], fig. 6(f). In the nanoscale IR spectra, the peak wavelength for Iris’ scan region (A) [201], fig. 6(f) appears shifted by about −12.5 cm^−1^ with respect to the forsterite standard [201], fig. 6(e). Their San Carlos forsterite standard appears to have a longer wavelength around 860 cm^−1^ (11.63 μm) compared with laboratory absorption spectra of ground forsterite at 893–897 cm^−1^ (11.2–11.15 μm) [152,155,210]. IR absorption spectra of forsterite are sensitive to the crystal shapes, e.g. ball-grinding versus hand-grinding tends to produce more spherical-shapes for crystals and shifts some resonances to shorter wavelengths [210] and rectangular-prism-shaped forsterite crystals fit cometary IR spectra better than spheres or ellipsoids [155]. IR absorption spectra of forsterite also are sensitive to the temperature [153]. In order to compare the nanoscale IR spectrum of Iris to laboratory absorption spectra of ground olivine of varying Fe-contents, we apply the observed shift between their standard and the Iris spectrum to derive a peak wavelength of 893–897 cm^−1^−12.5 cm^−1^=880.5 – 884.5 cm^−1^ (11.30–11.36 μm). This resonant peak position at 11.30–11.36 μm is near to {11.27, 11.33, 11.39 μm} that correspond to, respectively, {Fa40, Fa50, Fa80}[152,154]. The *Stardust* chondrule Iris composed of Fa35–Fa40 has an IR spectral peak (11.30–11.36 μm) that agrees with its Fe-content.

Thus, we note the wavelength of the olivine peak near 11.30–11.36 μm, which we have derived by translating between the nanoscale IR spectrum of Iris and laboratory absorption spectra of olivine powders, looks like Fe-rich olivine, giving us important clues of what to look for in remote sensing IR spectra of cometary comae. *Spitzer* spectra of comets can discern this shift. The far-IR resonances of crystalline olivine have larger shifts towards longer wavelengths with increasing *X*_Fa_ , so spectra in the 450–400 cm^−1^ (22–25 μm) range would be another good diagnostic for Fe-content of the olivine.

Iris has no hydrous silicate phases. Hydrous silicates (phyllosilicates) would indicate aqueous alteration, so their absence as well as the presence of glass argues against significant aqueous alteration (e.g. [101,107]).

Iris contains minerals with aluminium (oligioclase and Al in SiO_2_-rich glass) but no ^26^Al was detectable, so Iris was dated using the ^26^Al chronometer [15] at more than 3 Myr after CAI-formation (CAI-formation is ‘time-zero’ for solids in our ppdisc). Progress in the long-baseline ^206^Pb–^207^Pb dating oligoclase system reveals chondrules started forming at the CAI-epoch through approximately 3 Myr [8]. Thus, Iris having formed more than 3 Myr means Iris is a type II chondrule that was a late-formation ‘rock’ from our ppdisc [211]. The *Stardust* particle ‘Pixie’, which is a (forsteritic) FeO-poor crystalline silicate, has an age of greater than or equal to 2.6 Myr similar to the age of Iris, assuming ^26^Al was homogeneously distributed in the ppdisc [212]. In these cases, the absence of significant ^26^Al excesses may indicate a heterogeneous distribution of ^26^Al in the ppdisc which would mean the ^26^Al age-dating is not reliable; colloquially speaking, the jury is still out on heterogeneity versus homogeneity of ^26^Al.

Iris’ mineralogy has affinities with CR chondrites [15]. CR chondrites have ages near 3 Myr, which is towards the later epochs of chondrule-formation [213]. Thus, Iris may be a chondrule that formed in the late chondrule-forming regime in our ppdisc. T Tauri discs, which are analogue external protoplanetary discs, have inner disc dispersal ages of approximately 3 Myr and discs that can persist to 5–7 Myr [214,215]. In T Tauri discs observed by *Spitzer*, it was assessed that grains would grow to a few micrometre size and silicate dust crystalline fractions would increase and level out by approximately 1 Myr [216,217]. In our ppdisc, fine-grained materials persisted through the chondrule-forming epochs, because fine-grained materials exist in the matrix of carbonaceous chondrites along with type II chondrules or type II chondrule fragments. The chondrite record for our ppdisc indicates grains continued to grow and crystalline material continued to be generated through more than 3 Myr. Compared with external T Tauri inner discs observed by *Spitzer*, the submicrometre to less than or equal to 10 μm-size grains continued to evolve in our ppdisc to later times, later than the approximate 1 Myr perceived for grains grown in T Tauri discs and later than the typical inner disc dispersal ages of 3 Myr.

## 3. Kool: chondrule precursors in *Stardust* and chondritic porous interplanetary dust particles

In *Stardust* tracks, Kool particles are more frequent than chondrules so they are worthy of discussion. Kool are assemblages of submicrometre (≈0.5 μm) Na- and Ca-rich pyroxene together with FeO-rich olivine. Kool assemblages are characteristic of more than 50% of *Stardust* tracks (8/16 tracks in the aerogel collection media) [189]. In charge-balance substitutions in pyroxene, stoichiometric calculations show that the largest substitution for Ca would be a kosmochlor (NaCr_2_O_6_) component [218], figs. 15 and 20. Kool grains therefore are defined by Kosmochlor high-Ca-pyroxene occurring with Fe-rich olivine (FeO>10 wt%) [218]. Kool assemblages also have been observed in a handful of CP IDPs [218]. Kool assemblages have not been seen in chondrites but their abundance in *Stardust* and in CP IDPs suggests they were important components in the ppdisc [11].

Kool assemblages are hypothesized to be early generation chondrules (for equilibrated Kool) or chondrule precursors (unequilibrated, fine-grained Kool) [218]. MELTS models were applied to the Kool grain ‘Coki-B’ and the cooling of the melt fell out of thermal equilibrium at a temperature only 50°C lower than Iris [14]. In one-third of the Kool assemblages, there is more Na than Cr by up to a factor of 2. The Kool assemblages are delicate crystals that can lose their crystalline structure and become amorphous as well as lose their Na when examined by the electron beam in TEM [14] so care was taken to use low doses [218]. Almost every fine-grained Kool assemblage was associated with iron sulfides (e.g. FeS) [14]. In an igneous system like Stardust particle ‘Puki-B’, if the bulk composition was originally S-rich, then sulfur would remain in the melt until low temperature when sulfides would have formed. So, S-rich, Na- and Ca-rich bulk compositions typify Kool particles.

Type II chondrules, specifically porphyritic olivine (PO) chondrules, in unequilibrated ordinary chondrites (UOCs) have similar bulk compositions as Kool grains but the chondrules lack clinopyroxene phenocrysts. If Kool grains and type II PO chondrules in UOCs crystallized from melts of similar composition, then their crystallization sequences were different [218]. Note that the formation of kosmochlor found in *Stardust* samples appears to be at odds with its typical origin as a result of medium- to high-pressure metasomatism on the Earth [219]. The high Na-contents of Kool grains and of UOC type II chondrules are a mystery, as described in many articles (cf. [220]). Chondrite groups (UOC, CC, EC), CC chondrite classes (CO, CH, CM, CR, CV, CB, and CK) and chondrule textures (PO, PP, POP) are introduced in the electronic supplementary material, section (a).

## 4. Calcium–aluminium inclusions and ameboid-olivine aggregates, and oxygen isotopes (in brief)

Chondrules contain CAIs and AOAs. CAIs are high-temperature materials, often referred to as refractory inclusions and thought of as the first solids of the ppdisc [8]. The age of their formation represents ‘time zero’ for our ppdisc evolution because after that point they never reached high enough temperatures to be destroyed and removed from the meteoritic record. AOAs are melted mixtures of CAIs, forsterite (Fa0) and Fe,Ni metal [221]. Given their high temperatures of formation, CAIs and AOAs often are referred to as refractory inclusions. CAI-forming regions were notably ^16^O-rich [222]. LIME olivine are condensates from similarly early epochs of ppdisc evolution (the solar nebula phase) as when CAIs formed. LIME olivine are low-iron manganese-enriched forsterite [223].

*Rosetta* COSIMA searched for CAI grains in dust sampled from the coma of comet 67P and found one CAI-like grain [187].

CAIs were identified in *Stardust* samples [11,224–226]. *Stardust* CAIs are ^16^O-rich, with δ^18^O≈δ^17^O=−40‰ [224].^21^ A *Stardust* forsterite grain in track T112 has ^16^O-enrichments of approximately −40‰ (versus SMOW) and ‘possibly formed together with AOAs, via condensation’ [227]. A *Stardust* Al-rich chondrule fragment ‘Bidi’ that contains forsterite (Fa3), Al- and Ti-bearing clinopyroxene and anorthite (An97) is similar to AOAs and Bidi is mildly ^16^O-rich [189], fig. 15. Bidi is located in the same region of the three-isotope plot (δ^17^O versus δ^18^O) as one group of anhydrous IDPs with δ^17^O≈−5‰, where the other group of anhydrous IDPs is at δ^17^O≈−2‰ [228], fig. 4. Bidi and two other *Stardust* CAIs are compared with three refractory minerals from giant CP IDP U2-20GCP. Their bulk compositions as plotted in the Mg_2_SiO_4_–Al_2_O_3_–Ca_2_Si_4_ phase diagram and reveal that these six cometary particles fall on a line subparallel to an equilibrium condensation trend at *P*_tot_=10^−5^ atm≈10^−5^ bar [177], fig. 1 and [176], fig. 16. These materials represent type C CAIs and not the most refractory type A CAIs, suggesting that *Stardust* and this giant CP IDP sampled materials from ‘more evolved nebular reservoirs’.

All of the *Stardust* LIME olivines that have been analysed are ^16^O-rich, similar to CAIs [11]. LIME in *Stardust* samples are discussed in [12,189,229]. Besides LIME olivine, the osbornite grains found in *Stardust* CAIs probably represent the first condensates, having formed in an unusual C/O-enriched region of the solar nebula [11,230]. Osbornite had to form in very hot conditions in the inner solar system [231].

Although poorly determined, the abundance of refractory inclusions in the *Stardust* collection is on the order of 1% [11].

**Figure 5.**
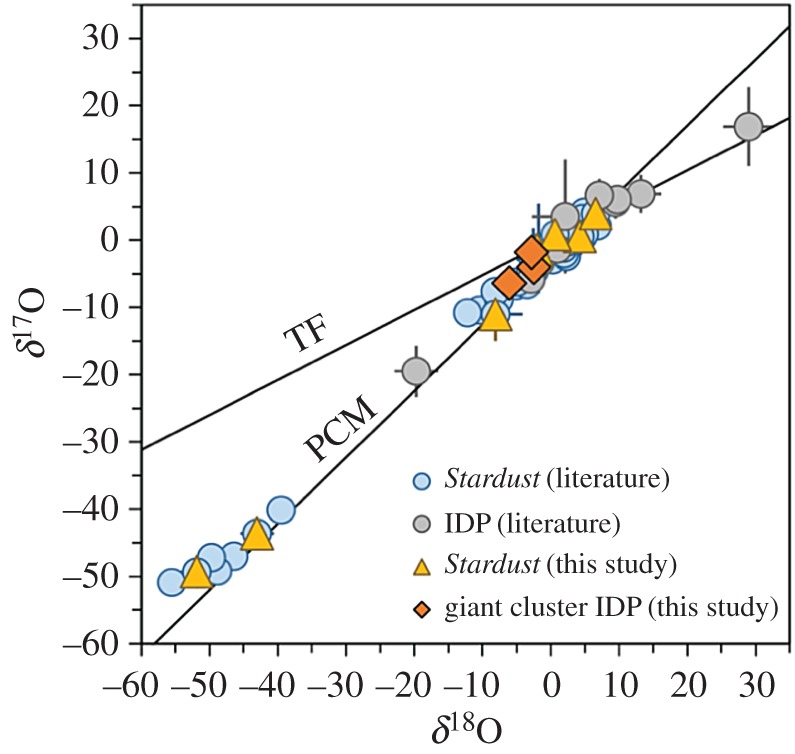
Oxygen three-isotope ratios of seven new (*Stardust*) Wild 2 particles and 3 GC-IDP particles. TF and PCM represent the terrestrial fractionation line and the primitive chondrule line (approx. slope 1 line), respectively. Literature date from [15,107,203,205,233,234] (reproduced with permission from [232], fig. 1 Copyright (2016), C. Defouilloy). (Online version in colour.)

**Figure 6.**
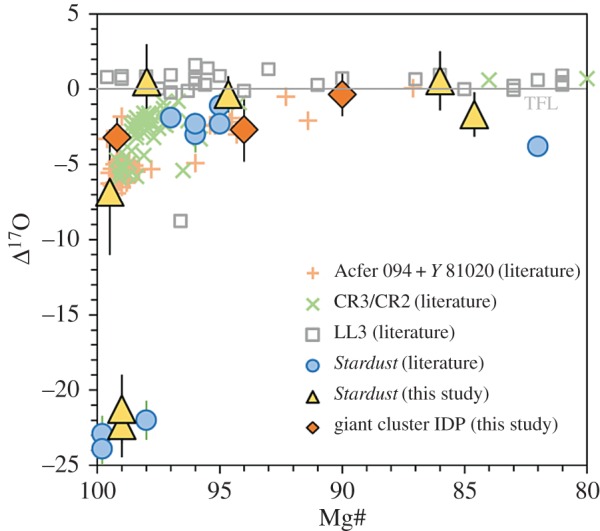
Relationship between Mg# (i.e. *X*_Fo_) and Δ^17^O in ferromagnesian (*Stardust*) Wild 2 particles and comparison to chondrules in primitive chondrites. Literature data from [203,211,233,234]. Chondrites data from [235–239]. See more details in [240] (reproduced with permission from C. Defouilloy [232], fig. 2. Copyright (2016)). (Online version in colour.)

Defouilloy *et al.* [232] report on ^16^O-contents in *Stardust* grains and in a giant CP IDP: two pyroxene grains are very ^16^O-rich with, respectively, δ^18^O=−51‰, −43‰ and Δ^17^O=−22.3‰, −21.3‰, and the giant CP IDP is in the same range as the ^16^O-poor *Stardust* particles with −6.1≤δ^18^O≤−2.5 and −3.2≤Δ^17^O≤−0.4, see [232] for the quoted uncertainties. Defouilloy *et al.* also show that ^16^O-rich is correlated with high Mg-content (figures 5 and 6). The link between oxygen isotopes and mineral chemistry in *Stardust* grains is discussed in detail in [240]. A few smaller (less than 2 μm) *Stardust* particles in the bulb of track C2052,74 have extreme ^16^O-enrichments, which do not correlate with *X*_Fa_ [211]. Larger corundum grains, which is a mineral in CAIs (1–5 μm) have ^16^O-rich compositions of (mean Δ^17^O=−22‰ with 15‰ standard deviation in δ^18^O [211], §7.3. In contrast to the *Stardust* CAIs and AOAs, *Stardust* chondrules have oxygen isotopes nearer to solar [211].

The oxygen isotopes are a full story in themselves [241], and we provide a brief synopsis (cf. [213], fig 1). The prevailing idea is that there were two O-isotopic reservoirs in the early solar system, ^16^O-rich and ^16^O-poor. The current ^16^O-content is due to the location of the material in the nebula, and its alteration (or lack thereof) history. ^16^O-rich material is found in its most concentrated form in some anhydrous chondrules and ^16^O-poor material is best sampled by grains that witnessed early oxidation/hydration reactions [242]. Type I chondrules typically are slightly more ^16^O-rich and type II chondrules slightly ^16^O-poor.

**Figure 7.**
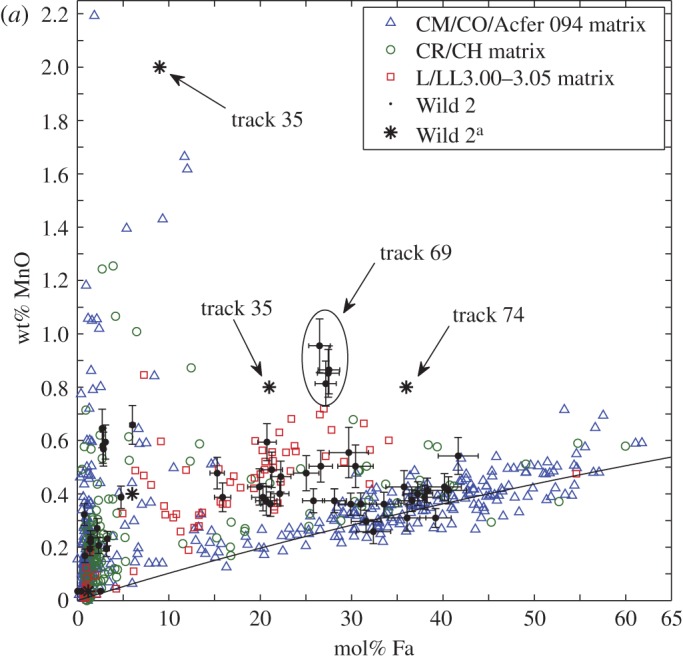
Mol% Fa versus MnO for olivine in isolated matrix grains and Wild 2. For reference, the solid lines through the origins delineate the CI ratio [261]. ^a^Compositions of the chondrule-like objects/fragments reported by Nakamura *et al.* [203] (tracks 35 and 108) and [15] (track 74) are included in the Wild 2 dataset, as well as one data point from [189] (track 10). (*a*) Chondrite types with similar olivine compositions are grouped together for comparison. (*b* [not shown]) The majority of FeO-rich olivine from Wild 2 is shown as overlapping two distinct compositional fields; one defined by UOC and CR/CH isolated matrix grains, and one defined by CCs (includes CI, CM, CR, CH, CO, CV-reduced, and Acfer 094 chondrites, but excludes CV-oxidized isolated matrix grains). Other unusual MnO-enriched, FeO-rich olivines from our data set are shown for comparison (see text of Frank *et al.* [12] for this discussion) (reproduced with permission from [12], fig. 8. Copyright © 2014 Elsevier). (Online version in colour.)

The favoured explanation for two reservoirs is CO self-shielding, which was hypothesized by Young & Russell [243]. How the oxygen isotope reservoirs became distinct has been modelled based on the hypothesis that CO with heavy O was more prevalently dissociated in regions where gas-grain chemistry occurred on cold grain surfaces compared to CO with the ^16^O-isotope. In molecular clouds, CO is the next most abundant molecule compare with H_2_. C^16^O is so much more abundant than C^17^O and C^18^O that the path lengths for UV photons capable of dissociating CO are short for C^16^O compared with C^17^O and C^18^O. UV photons that can dissociate C^17^O and C^18^O can penetrate deeper into molecular clouds. Where UV photons can penetrate, C^17^O and C^18^O become dissociated and the ^17^O and ^18^O become incorporated into O-heavy water via reactions with abundant gas phase H_2_ on cold grain surfaces.^22^ Many references discuss O-isotopes in chondrules and matrix grains because of the interest in how the oxygen reservoirs contributed to the mineralogy. One might think that if high oxygen fugacity regions existed early in ppdisc evolution, then perhaps the Fe-rich olivine grains could be found to be ^16^O-rich; so far, only Fe-poor olivine is ^16^O-rich. CR chondrites have a handful of Fe-poor ‘relict’ grains in the cores of Fe-rich type II chondrules that have slight ^16^O-enrichments compared with CAIs and that are in the same range as some type I chondrules [244], fig. 7. CR oxygen isotopes are discussed in [244,245]. ^16^O-rich CAIs and AOAs in CH chondrites are shown in [246], fig. 15.

## 5. Diversity of olivine in chondrules, *Stardust* and giant chondritic porous interplanetary dust particles

### (a) Synopsis

Frank *et al.* [12] present a comprehensive study of the cores of *Stardust* olivine grains (aerogel track terminal particles) and similarly sized (5–30 μm) matrix olivine grains from the least metamorphosed carbonaceous chondrites and ordinary chondrites. Frank *et al.* compares the Fe and Mn contents, as well as the Ca and CrO contents, as functions of Fe-content (wt%) or *X*_Fa_ (mol%). Frank *et al.* demonstrate that *Stardust* Fe-bearing olivine grains have a more diverse Fe–Mn relation than the full range of Fe–Mn relations spanned by fine grained matrix in all carbonaceous chondrite classes.

From the comparison of Fe–Mn relationships between cometary samples and asteroidal samples (chondrites), the conclusion is reached that cometary materials sample a broader reservoir than any meteorite sampled to date, with possibly the exception of the meteorite Kaidun [247]. The inference is that there was a greater diversity of materials transported to the ppdisc regime where comet 81P accumulated compared with the diversity of materials that accumulated in any single chondrite class or asteroidal parent body. Carbonaceous chondrites formed from materials from 1 AU and on short enough time scales for distinct differences between reservoirs to be recorded/preserved. More recent work on a greater number of *Stardust* olivine grains and on one or more giant CP IDPs, of probable cometary origin, also show that *Stardust* and a few giant CP IDPs have this same incredible diversity in their Fe–Mn relations [177,179].

Based on these few cometary samples that have been extraordinarily well studied, it appears that a handful of comets (81P and the cometary sources for the giant CP IDPs) have a similar high diversity of materials, implying that these comets sampled ppdisc regimes that had similar compositions resulting from similar radial transport processes/efficiencies from a variety of type II chondrule-forming reservoirs. The similarity of extreme ranges of properties of primitive cometary matter is very important. Of lesser significance is the possible implication that these comets formed at similar late times in our ppdisc evolution, keying off the *Stardust* particle ages of greater than or equal to 3 Myr for type II chondrule Iris and greater than or equal to 2.6 Myr for refractory igneous grain ‘Pixie’ (§2), as well as three other *Stardust* particles [211] including CAIs ‘Coki’ [225], ‘Coki-B’ and ‘Inti’ [248]. CR chondrites formed at similar ppdisc ages of 3 Myr [213,249] but CR olivine does not span the full range of compositions shown by olivine in *Stardust* and in the giant CP IDPs under discussion. The late formation of these comets is a sufficient but not necessary cause for the diversity of Fe–Mn relations because of (i) the age-dating is based on a contestable assumption of homogeneous distribution of ^26^Al in the disc and (ii) there may be more chondrites to discover such as Kaidun, which appears to have incorporated multiple chondrite reservoirs.

Carbonaceous chondrite classes have specific patterns in their Fe–Mn relations, which is why the extremely broad range of Fe–Mn relations for *Stardust* grains is so interesting. We review aspects of chondrites, composed of chondrules and matrix (electronic supplementary material, section (a), §5c). The Fe–Mn relations for a few chondrite classes are discussed in detail, with the aim of facilitating the impact of the importance the Fe–Mn relation on comet origins. In this process of this discussion, we also highlight some of outstanding mysteries of chondrule formation.

### (b) Mysteries of type II chondrule formation

The formation of Fe-rich olivine and of type II chondrules holds five mysteries: How is the high oxygen fugacity, which is needed to form Fe-rich olivine, attained? How/why is the Fe/Mn ratio maintained or diminished during the melting event? How is Na concentrated or retained? How is the depletion pattern (electronic supplemental material, section (c)) manifested, where by type I versus type II chondrules and matrix, each possessing widely different Mg- and Fe-contents, sum together to almost CI-composition (to almost to solar composition)? What are the chondrule-forming mechanism(s) for heating and rapid cooling?

High oxygen fugacities can result from high dust/gas ratios and from the dissociation of water. The melting of H_2_O ice [238] presumably occurred in a ppdisc regime near the water ice evaporation/condensation front (the ‘snow line’) or via infiltration of cometesimals interior to the evaporation front [250,251]. Particle growth and inward drift dominate in the first 2×10^5^ yr, such that just outside evaporation fronts there are enhancements in solids that are created when drifting vaporized material diffuses back outside the evaporation front, re-condenses on small grains and advects or diffuses some (greater) distance before becoming accumulated into a larger particle [252]. At later disc ages, typical of the T Tauri phase, outward transport of dust also occurs around the mid-plane [253] as well as above the mid-plane (particle trajectories look like a random-walk up/down and outward) [28]. Pre-shock heating coupled with the passage of large-scale shocks in the ppdisc may concentrate the right size distribution of dust to produce chondrule cooling rates [254]. The oxygen isotopes play in important role in deciphering type II chondrule formation, as summarized by Gainsforth *et al.* [14]: ‘Oxygen isotope measurements of type II chondrules often show heavier O than type I chondrules [235,238]. In some models, production of heavy oxygen is connected to the presence of water and may even be a tracer for water in the early solar system [238]. ^26^Al isotopic measurements show that type II chondrules typically formed contemporaneously or later than type I chondrules [15,255,256]’. Type II chondrules require higher gas/dust enrichments than type I chondrules, as well as higher pressures to explain Na-enrichments. However, lower gas densities are expected as the ppdisc ages. ‘Resolution of this mystery may be related to the formation of the planetesimals themselves, if these high densities are produced by the planetesimals in the form of shock waves [257]’. The overall picture of the importance of type II chondrules is that type II chondrule formation tracks the reservoirs of enhanced oxygen in the ppdisc via dust enrichment and possibly through the incorporation of a ^16^O-poor water [14].

As we review the basics of type II chondrule properties and the mysteries of their formation, we mull over the following speculation: Could *Stardust* have type II olivine material because these were the smaller/smallest particles of chondrule-formation that were ‘bled off’ the chondrule-forming ppdisc region by aerodynamic size-sorting and outward radial transport? Could the loss of fine-grained material to the outer disc be a contributing factor to the temperature-selective loss of volatile and moderately volatile elements from the matrix reservoir (the depletion pattern electronic supplementary material, section (c))? Type II chondrule/fragments are much smaller than type I chondrules, perhaps partly because high-Fe silicates are more brittle and/or perhaps high-Mg silicates preferentially aggregated to mm-size and were not turbulently lofted to significant heights above the midplane [258] prior to melting. Type I chondrules of millimetre size and larger are much harder to transport radially outwards in the ppdisc compared with smaller particles (10–30 μm size), which are in turn harder to transport compared with the ‘smallest’ grains (μm size) [27,28,258,259].^23^ Perhaps selective loss of fine-grained materials may have occurred from the non-type I reservoir, i.e. from the matrix material. The story potentially would be: the ‘lost matrix material’, which is typified by type II chondrule fragments, is found in (some) comets as Fe-rich olivine grains, due to preferential transport of the fine-grained fraction.

### (c) Fe–Mn relation for olivine

The Fe–Mn relation for olivine in type II chondrules or chondrule fragments shows that each chondrite group appears to sample a distinct reservoir with specific geochemical characteristics. In olivine, Mg, Fe and Mn are oxidized (bound with O) so the Fe–Mn relation is represented equivalently in diagrams that plot MnO (wt%) as a function of FeO (wt%), Mn (atomic formula units, afu) as a function of Fe (afu), or MnO (wt%) as a function of Fa (mol%), where definition for Fa and Fe are in §2.

**Figure 8.**
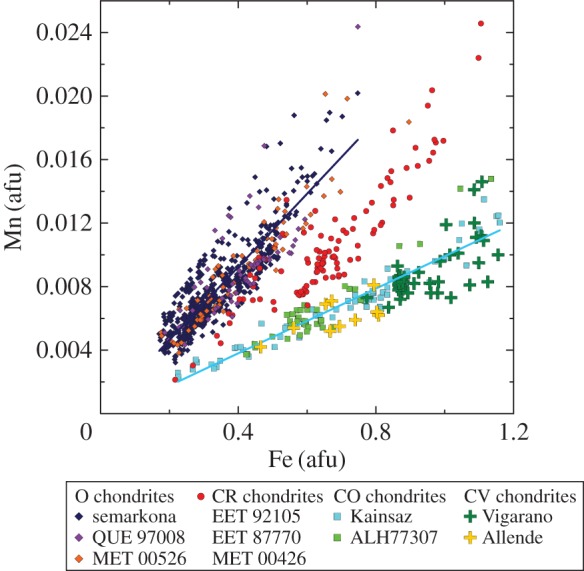
Characteristics of type IIA porphyritic olivine chondrules. Mn versus Fe contents of olivine in typeII chondrules from different chondrites. After [262] with additional, previously unpublished, data (R. Jones) for Vigarano and Allende. The upper line is the best fit to all OC data, and the lower line is the best fit to all CO data (reproduced with permission from [263], fig. 3b. Copyright © 2012 John Wiley & Sons, Inc.) (Online version in colour.)

**Figure 9.**
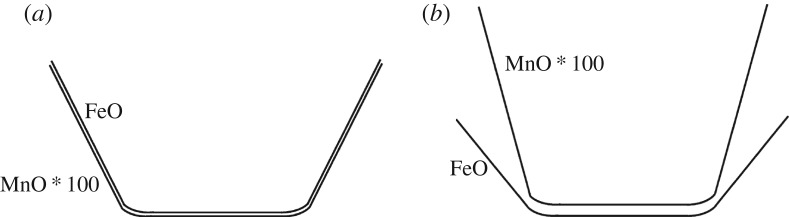
(*a*) Schematic zoning profile for a CO type IIA chondrule olivine. The Fe/Mn ratio stays constant during olivine crystallization. (*b*) Schematic zoning profile for a CR or OC type IIA chondrule olivine with a negative trend in the molar Fe/Mn versus Fe/Mg diagram as shown in (*b*) (not shown) (reproduced with permission from [262], fig. 11(c,d). Copyright © 2011 John Wiley & Sons,Inc.).

Frank *et al.* [12] report their study focused on comparing equivalent-sized (5–30 μm) matrix grains from carbonaceous chondrites (CC) and unequilibrated ordinary chondrites (UOC) with *Stardust* grains from comet 81P. In figure 7, we show their plot of MnO (wt%) as a function of Fa (mol%) [12], fig. 8, see also [12], figs. 2, 8, 9. *Stardust* olivine grains span a wide range of Fe contents from Fa0–Fa44 (with one Fa100 not shown that has 5.1 wt% MnO, which is extraordinarily high). The CI-line is shown for reference, which is the line that passes through the origin that represents a constant ratio of Mn/Fe equal to CI chondrites (CI=solar composition). *Stardust* olivine grains are in regions of the plot occupied by multiple chondrite classes: Fa25–Fa44 mol% are in the range of matrix olivine grains from CO, CM and Acfer 094 (C-ungrouped) chondrites; Fa15–30 mol% with slightly higher Mn contents are in the range of matrix olivines from CR and CH as well as from L/LL3.00–3.05; Fa ≤ 7 are in the range with type I chondrules and low-Fe matrix grains from many chondrite classes. *Stardust* particles identified by asterisks and track numbers are specific *Stardust* chondrules because of their complex mineralogy. (‘Track 74’ is the *Stardust* chondrule ‘Iris’ (§2, [14]. Torajiro is the one of the two ‘Track 35’ particles, the one that is further from ‘Track 74’ near Fa=8 (mol%).) The *Stardust* chondrules ‘Iris’ and ‘Torajiro’ appear to have higher values of MnO (wt%) than *Stardust* olivine grains shown by Frank *et al.* More recent analyses of a selection of comparable numbers of *Stardust* olivine grains reveals *Stardust* olivine grains well-populate the whole range from the left axis to the CI-line [11,179]. In the combined samples of Frank *et al.* and Brownlee *et al*., *Stardust* chondrules do not stand out of the crowd of *Stardust* olivine grains in the Fe–Mn diagram.

Figure 8 [263], fig. 1 shows the Fe–Mn relation for type II olivine in specific meteorites representing diverse chondrite groups (UOC=O, and CCs=*CR*, CO, CV). Note that only type II chondrules are shown in this plot of Mn (afu) versus Fe (afu). A main point is that chondrite classes are distinguishable through the Fe–Mn relation in their type II olivine. The large number of points represent multiple measurements of (different) olivine grains within a much smaller number of chondrules and chondrule fragments. Two lines are fitted, one line through all the UOC measurements with a slope of 0.023 and one line through the CO and CV with a slope of 0.010. The inverse slopes correspond to the values of Fe/Mn ratio ≈44 for UOC and Fe/Mn ≈ 100 for CO chondrites [262], fig. 4. The CR chondrites appear to occupy a broader range than spanned by CO/CV, extending well into the UOC region [263,264]. Different chondrite classes occupy distinct regions of the Fe–Mn plot because they have distinguishable Fe/Mn ratios (inverse slopes on that plot). It is important to note that the choice of sampling of type II chondrule olivines cited in these references authored by meteoriticists [244,249,262–264] are different than the sampling of Frank *et al.* because the former made multiple measurements of type II chondrule olivine, e.g. to establish zoning patterns, compared to Frank *et al*., who made measurements of only the cores using a 1 μm focused beam and only of specific sized (5–30 μm) matrix type II olivine grains. The trends are similar between multiple measurements of type II chondrules and individual chondrite matrix olivine, which is one reason that chondrite matrix olivine grains are considered to be type II chondrule fragments.

### (d) Zoning patterns

Zoning patterns show that changes in the Fe-content of the olivines, cf. [262], fig. 1, and changes in the Fe/Mn ratios occur during crystallization, which have implications for the physical conditions during chondrule formation. Zoning patterns for type II olivine are diagrammed in figure 9 [262], fig. 11(c,d): towards the outer edges of a CR type II chondrule, either FeO and MnO increase at the same rate yielding a constant Fe/Mn ratio (left, ‘c’) or MnO increases at a greater rate than FeO (right ‘d’) yielding a decreasing Fe/Mn ratio towards the edge of the chondrule. Often the zoning trends are shown on plots of Fe/Mg (mol%) versus Fe/Mn (mol%) [262].

**Figure 10.**
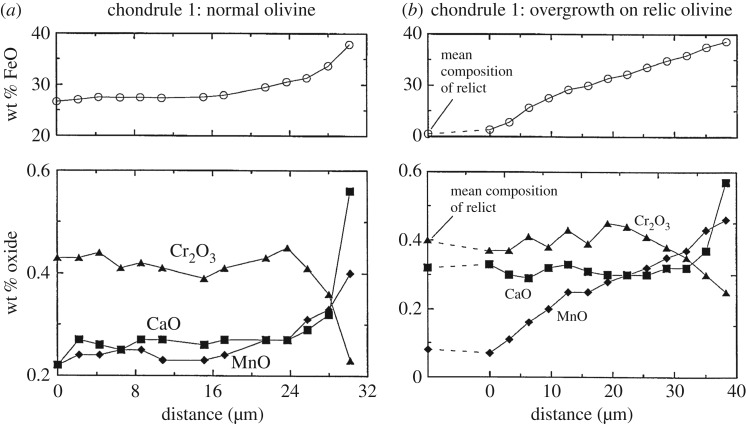
Zoning profiles for olivine grains from (type IIA, porphyritic olivine (PO)) chondrule 1. Positions of profiles are illustrated in [51], fig. 1h. (*a*) Profile from core to edge of a normal zoned olivine grain, showing characteristics of growth during igneous crystallization from the chondrule melt. (*b*) Profile across the boundary between relic grain 1 and its igneous overgrowth. The mean composition of the core of the relic grain 1 is indicated on the left of the plot(reproduced with permission from [51], fig. 3. Copyright © 2000 John Wiley & Sons, Inc.).

First, we consider the case of Fe/Mn constant during crystallization as in the trend for CO type II olivine. This zoning pattern with Fe/Mn constant means that during crystallization from melt neither MnO nor FeO are ‘lost’ and the igneous system is ‘closed’. The zoning pattern in individual chondrules translates to a particular chondrule spanning a line segment of constant Fe/Mn. Since all CO type II olivine have similar constant Fe/Mn ratios, in the Fe–Mn diagram there is a fairly tight line for CO. For example, the zoning in a normal type II olivine grain, designated ‘Chondrule 1’ in figure 10 spans Fe ≈ 26–38 wt% and Mn ≈ 0.24–0.4 wt% [51], fig. 1, left. The zoning translates to Fe/Mn ≈ 110–95, which falls nicely along the CO line in the Fe–Mn plot. Some type II chondrules contain in their cores ‘relict’ FeO-poor olivine and the overgrowth of FeO-rich and MnO-rich material on relict grains typically reaches the value of the host Fe/Mn olivine at the surface. Fe-rich olivine overgrowth on FeO-poor ‘relict’ forsterite is shown for a second olivine in ‘Chondrule 1’ in ALHA77307 [51], fig. 1, right. Also, see [262], fig. 6 where two relict grains in the chondrite Kainsaz CO3.2 are identified in the Fe–Mn plot because the core relict grains are not as Fe-poor as typical ‘relict’ grains.

Second, we consider the other zoning pattern for type II chondrules where MnO/FeO increases towards the surface and inversely the Fe/Mn ratio decreases. This zoning pattern means that either MnO is added by condensation or FeO is lost to the gas by reduction. Reduction is the change from Fe^+2^ bonded in FeO to Fe^0^, typically stimulated by a low oxygen fugacity (low available oxygen in the gas phase), with subsequent loss of Fe metal to the gas phase (sometimes described as loss of tiny Fe-metal blebs). Fe metal is rare in type II chondrules so FeO reduction is not a favoured explanation [244,262,264]. Rather, MnO addition by condensation is favoured as an explanation for the zoning pattern. Zoning contributes to explaining the UOC region appearing broader than the CO region of the Fe–Mn relation because zoning in olivine in individual UOC type II chondrules yields multiple line segments each with slightly steeper slopes than the correlation line (Fe/Mn ≈ 44), which manifests as a slight vertical smearing of the correlation [262,263]. That is, zoning tends to vertically smear the Fe/Mn correlation.

The fact that zoning causes slight modifications in the Fe/Mn ratio means that zoning does not move chondrule olivine out of regimes corresponding to that chondrite class. Hence, the discrimination between different carbonaceous chondrite classes in the Fe–Mn plot are due to significant differences in precursor composition or oxygen fugacity, meaning that there are distinguishable reservoirs and physical conditions that pertain to distinct chondrite classes [262,263]. The addition of MnO (by condensation) during crystallization is the favoured explanation for zoning in CR type II chondrules (§5e).

### (e) CR chondrule olivine

Next, we embark on explaining the wide range of the Fe–Mn relation that is occupied by type II olivine grains in CR chondrites because CR and *Stardust* both occupy broad regions in the Fe–Mn plot. A note of interest is that CR chondrules have been age-dated at ∼3 Myr and so represent chondrule formation in the later-stages of our ppdisc if we consider age-dating of all chondrules [213] as well as if we take the typical age of dispersal of inner discs of analogue T Tauri systems to be *ca* 3 Myr (§2). Also, the *Stardust* chondrule Iris has been age-dated at *ca* 3 Myr and Iris is most similar to chondrules from CR and CB chondrites [211], although Iris’ higher Na, Ca and Al means Iris is unlikely to have formed precisely among CR chondrites [15].

Schrader *et al.* [244] summarize the properties of CR chondrites [264], table 3. CR chondrites are predominately composed of chondrules (65.9 vol%), fine-grained matrix (33.7 vol%), and CAIs and AOAs (0.4 vol%) (§4). The most dominant chondrule type is type-I chondrules at 95.8% of the chondrule population. In stark contrast, type-II chondrules constitute 3.5% [249], table 1, with the remaining 0.7% made up of aluminium-rich chondrules and agglomeratic-olivine chondrules [244]. One hundred and forty-one CR type II chondrules were studied, which accounts for around 2 vol% of bulk CR chondrites or around 4% of the total chondrule population by volume. The breakdown of type II olivine textures and minerals are as follows: 7.8% chondrules, 41.1% chondrule fragments, 40.4% fragments and 10.6% grains. In total, 55% PO and 38% POP and 7% other compositions/textures (cf. electronic supplementary material, section (a)). In CR2 chondrites (Renazzo), the bulk chondrite Mg/Si ratio is CI (solar) so there is a mass balance between Mg-rich type I chondrules and Fe-rich matrix [265], fig. 7. The matrix contains Fe-rich matrix grains that are considered type-II chondrule fragments. However, the matrix also contains enhanced volatile and moderately volatile elements. CR2 show strong evidence for genetic complementarity between matrix and type I chondrules [264,265] (electronic supplementary material, section (b)). CR chondrites frequently contain relatively high concentrations of presolar grains and primitive organics, suggesting that CR matrix escaped some parent body processing [266].

Schrader *et al.* [264] state, ‘The dominance of type II chondrule fragments, igneous fragments, and mineral grains over intact chondrules [264], table 3a suggest mechanical processing’. In other words, a ppdisc environment with a high dust/gas ratio (approx. 10^4^) not only produces the high log(*f*_O_2__) needed to form type II olivines (§2) but also is conducive to grain–grain collisions. Moreover, FeO-rich mineral grains are considered chondrule fragments, because their compositions and O-isotopes are indistinguishable from type II chondrules [264]. Note that this supposition that FeO-rich matrix grains are chondrule fragments is applied to matrix grains in various chondrites as well as to *Stardust* olivine grains [11,12,14,15,203].

**Figure 11.**
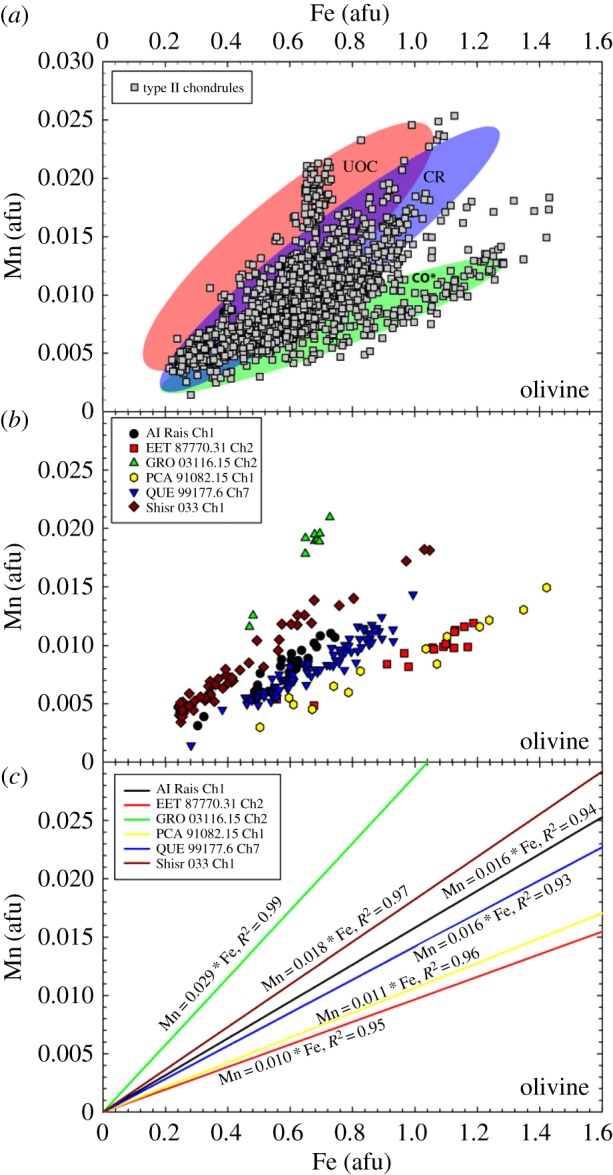
(*a*) Fe versus Mn (afu) for FeO-rich olivine from type II chondrules of this study compared to the ranges for UOC, and CR and CO chondrites (*) determined by [262]. (*b*) Fe versus Mn (afu) for select type II chondrules with a highnumber of analyses. This plot shows the spread of compositions represented by the CR chondrites, and trends represented by individual chondrules. (*c*) Linear regressions for chondrules shown in (*b*), showing distinct slopes and high correlation coefficients (R) for each chondrule. (*d*) Fe versus Ca (afu) and (*e*) Fe versus Cr (afu) for the same chondrules shown in (*b*) and (*c*). Individual chondrules have trends/groupings with Fe versus Ca and Fe versus Cr (reproduced with permission from [264], fig. 8. Copyright © 2015 John Wiley & Sons, Inc.). (Online version in colour.)

For CR type II olivine in figure 11, there is no correlation in the Fe–Mn diagram for all measurements (figure 11*a*; correlation coefficient *R*^2^≈0.4). Individual chondrules with many measurements, however, show tight trends in the Fe–Mn relation (*R*^2^≈1, figure 11*b*,*c*). Distinguishable slopes indicate different Fe/Mn ratios (Fe/Mn ratios=inverse of slopes, figure 11*c*). The lack of a global trend but strong evidence for trends in individual chondrules implies that each chondrule was an independent igneous system [264].

**Figure 12.**
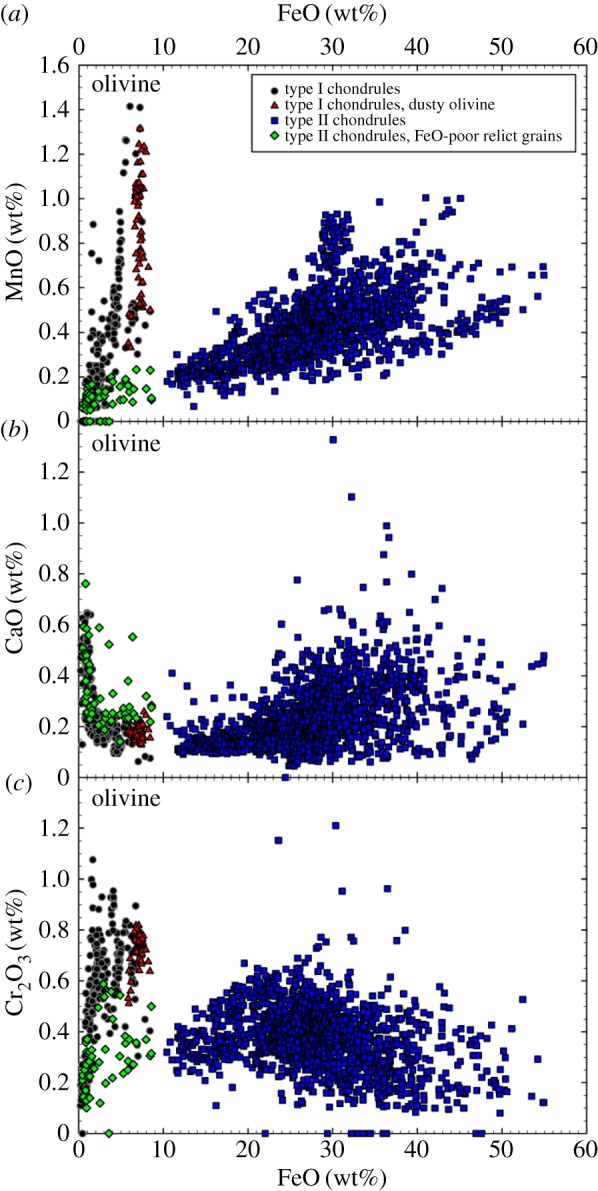
Major- and minor-element composition of olivine in 44 type I and 139 type II chondrules obtained via EMPA (electron microprobeanalyser). (*a*) FeO versus MnO. (*b*) FeO versus CaO. (*c*) FeO versus Cr_2_O_3_. (all wt%). Data points along the *x*- and *y*-axes have values which are below the detection limit of the EMPA [264], table 4 (reproduced with permission from [264], fig. 7. Copyright © 2015 John Wiley & Sons, Inc.). (Online version in colour.)

Four types of olivine grains are shown in the Fe–Mn plot for CR chondrites [264] (figure 12): type II chondrules (FeO>10 wt%), type I chondrules (FeO<10 wt%), relict ‘dusty’ FeO-rich grains in FeO-poor type I chondrules, and ‘relict’ FeO-poor grains in FeO-rich type II chondrules. CR ‘relict’ grains do not have low enough FeO to be classified as LIME. LIME particles are thought to be the first condensates and are defined by Fe/Mn<1 and *X*_Fo_=Mg(Fe+Mg)>0.99 [223]. LIME particles are found in *Stardust* [189], fig. 35, which is one of the distinctions between CR olivine and *Stardust* olivine. LIME olivine were introduced in (§4).

The hypothesis of Schrader *et al.* [264] for the formation of CR chondrites is that type I and type II formed from similar precursor materials but were subjected to heating in significantly different oxygen fugacities that were facilitated by both different amounts of carbon—a reducing agent—and different amounts if ^16^O-poor water ice—an oxidizing agent. This hypothesis is based on assessments that can be drawn from the Fe–Mn relation for CR chondrites (figure 12*a*) [244,264]. We itemize these points because they serve as a template for examining and interpreting the Fe–Mn relation, i.e. for looking at the plots of MnO (wt%) versus FeO (wt%) or MnO versus Fa (mol%) for CR olivine as well as for *Stardust* olivine.

*Gap*. There is a gap or change of behaviour between type I and type II olivine at FeO ≈ 10 wt%.
*Similar wide range of MnO*. MnO-content of chondrule olivine ranges from 0–1.0 wt% for type II and the majority of type I. A few type I reach to MnO 1.4 wt%, which is the upper range for matrix olivine for most chondrite classes shown by Frank *et al*., with the exception being CM matrix olivine (*X*_Fa_≈10 mol% that reaches to 1.7 wt% [12], fig. 2.
*‘Relict’ grains are not type I chondrule fragments*. ‘Relict’ olivine grains are FeO-poor (Mg-rich forsterite) that are found preserved in cores of type II chondrules. ‘Relict’ olivine grains have little MnO. ‘Relict’ olivine grains are not fragments of type I chondrules because type I chondrule olivine have a wide range of MnO.
*Type II olivine not dependent on the presence/absence of ‘relict’ grains*. Type II chondrules with relict grains and without relict grains plot in the same region of the Fe–Mn diagram [244]. Type II olivine with relict grains have only barely increased Fe/Mn ratios in their zoning patterns [264].
*Type II zoning due to MnO addition*. The zoning pattern in type II chondrules, i.e. negative slopes on an Fe/Mg versus Fe/Mn plot (§5d), probably was due to MnO addition by condensation and not due to the loss of FeO by reduction [244,262,267,268]. Reduction of FeO to Fe metal under low oxygen fugacity conditions is unlikely because of the low abundance of Fe metal in type II chondrules. Some type II olivine show a flat zoning pattern with a constant Fe/Mn ratio, so there was no change in Fe or Mn during crystallization.
*Type I zoning due to FeO reduction to Fe metal*. Type I chondrule olivine shows stronger zoning patterns (decrease in the Fe/Mn ratio) than type II olivine. The zoning is attributed to the loss of FeO. There are ‘dusty’ olivine grains in the cores of some type I chondrule olivine. Formation of ‘dusty’ olivine has been experimentally reproduced by lowering the oxygen fugacity via carbon combustion to CO or CO_2_ [269]. Low oxygen fugacity also supports the high abundance of Fe metal in CR type-I chondrules; Fe may be expelled as metal blebs during chondrule melt-crystallization.
*Type I are not reduced type II*. No correlation is seen in type II between chromium (Cr_2_O_3_) and FeO (figure 12*c*). However, type I chondrule olivine has a strong positive trend of increasing chromium with increasing FeO (figure 12*c*). Therefore, conversion from type II to type I, or *vice versa*, is not favoured.
*Type II are ^16^O-poorer than type I*. All FeO-poor ‘relict’ grains contained in the cores of type II olivine are ^16^O-rich and are either completely or partially surrounded by relatively ^16^O-poor FeO-rich olivine. Thus, type II formed by the addition of oxygen probably derived from ^16^O-poor water.

From these assessments of the Fe–Mn relation, Schrader *et al.* [244,264] hypothesize CR type I and type II chondrules formed from the same reservoir (‘model b’ in electronic supplementary material, section (c)). Both types of chondrules formed from the same reservoir but under different oxygen fugacities determined by significantly different abundances of carbon and of ^16^O-poor water: ‘Therefore, we suggest that both the abundance of ice and reduced carbon that accreted with each chondrule precursor contributed to their individual log( *f*_O_2__). We suggest that the precursors of type-I chondrules contained more reduced carbon than type-II chondrules, creating an overall reducing environment. … In contrast, the type-II chondrule precursors had less reduced carbon and the accreted ice created an oxidizing environment upon melting. Therefore, each chondrule precursor may have accreted a similar abundance of ice and interacted with the same 16O-poor gas reservoir during melting, but contained different abundances of reduced carbon’ [244], §4.4.2.

Paraphrasing Schrader *et al.* [244]: distinct compositional relationships among individual chondrules imply chondrules acted as individual igneous systems that formed (i) under distinct oxygen fugacities (log( *f*_O_2__)), (ii) from varied precursors (i.e. different initial abundances of Fe and Mn), (iii) at different cooling rates, although we note that this explains only small variations in the Fe–Mn relation [262] and/or (iv) experienced either complete or incomplete re-condensation as evidenced by MnO addition in type II and by FeO loss or FeO conversion to Fe metal in type I. For chondrules from the CR chondrites, these conditions were as varied as the entire range of conditions under which chondrules from both the UOCs and COs formed. This is the same conclusion as reached for *Stardust* olivine [12,179,270].

Carbon is a major part of the scenario to form CR but carbon is not mentioned as a detected component. Carbon was detected in the least-altered component of the matrix of Paris, a CM2, along with GEMS-like amorphous silicate [92]. Paris type II olivine has a wider range of Fe-contents than CR [271]. The textures of GEMS-like amorphous silicates are easily destroyed by parent body metamorphism. Thus, Paris provides an exciting potential link to primitive materials, and may offer more conversation about primitive materials that are so abundant in comets and matrix materials of carbonaceous chondrites. In CR, the formation of type II chondrule olivine is inferred to be associated with less available carbon, which is a reducing agent. *Stardust* has notably less carbon than is typical for anhydrous IDPs and other cometary dust reservoirs [11]. It is interesting that *Stardust* has both abundant type II chondrule olivine and little elemental carbon, being that both correspond to hypothesized type II chondrule formation conditions.

## 6. UltraCarbonaceous Antarctic micrometeorites

UltraCarbonaceous Antarctic micrometeorites (UCAMMs)^24^ offer a different view into cometary materials compared with type II chondrule fragments from *Stardust* and from the least-altered chondrites. UCAMMs have substantially more carbonaceous matter and more S-bearing matter, which occurs as FeS grains and nano-size FeS (nFeS) in GEMS [272]. Olivine and pyroxene compositions nearly reach the wide span of FeO-contents of olivine matrix grains in *Stardust* and in type II chondrule/chondrule fragments but the Ca-poor pyroxenes and olivines with *X*_Fa_≤15 mol% dominate by number. UCAMMs have high carbon contents [272], trace presolar matter [273] and ‘early’ nebular material via ^16^O-rich carriers [274].

Three well-studied UCAMMs are discussed [195,272–276]. Their cross-sectional sizes are about 50 μm×80 μm. UCAMMs contain assemblages or clusters of submicrometre silicate crystals (0.05–0.5 μm, with a trend for greater frequency of smaller sizes). The low-Ca pyroxenes and olivines are most abundant mineral, with pyroxenes out-numbering olivines. Fe–Ni sulfides are the second most abundant mineral.^25^ For context, 15 wt% of comet Halley’s refractory dust was FeS and it was more abundant amongst the more massive particles [172]. The assemblages shown in [272] are 1–2 μm across and their submicrometre silicates share similar Mg/Fe ratios, whereas the Mg/Fe ratios vary between assemblages. The grain boundaries of the mineral crystals often are cemented by an SiO_2_-rich amorphous phase or SiO_2_-rich glass, which is depleted in Fe, Ni and S by a factor of 3–5 from CI. Distinct from this interstitial silica-glass are domains (blobs) of amorphous silicates with embedded nano-phase FeS (nFeS), which are remarkably similar to GEMS in anhydrous CP IDPs. GEMS, glass with embedded metal and sulfides, are considered to be the best analogues for inherited ISM silicates [18,23,75]. In UCAMMs, GEMS have diameters from 0.07 to 0.35 μm with an average size of 0.18 μm. Compared with 239 GEMS in nine anhydrous IDPs [81] and to 42 GEMS in five CP IDPs [78], the 47 GEMS in these three UCAMMs [272], fig. 9 have a similar bulk average composition (within 2×CI) but have a narrower range of Fe and Si in the Si–Fe–S ternary diagram and they fall in the S-rich area of the Mg–Fe–S ternary diagram [278]. GEMS in UCAMMs have abundant nFeS but little nFe. UCAMMs have a significant amorphous silicate component in the GEMS, and a crystalline-to-amorphous ratio of at least 25±3%. The UCAMMs’ bulk compositions are somewhat depleted with respect to CI but still within a factor of 2 of CI [272], fig. 4. On submicrometre scales, however, rare polycrystalline regions in UCAMMs have significant excursions from CI compositions: one polycrystalline assemblage has Mg-rich pyroxenes with Fe–Ni metal poikilitically enclosed in it and its bulk composition is within a factor of 2 of CI except for Fe, Ni and S that are depleted by factors of 3–5 [272], fig. 10.

**Figure 13.**
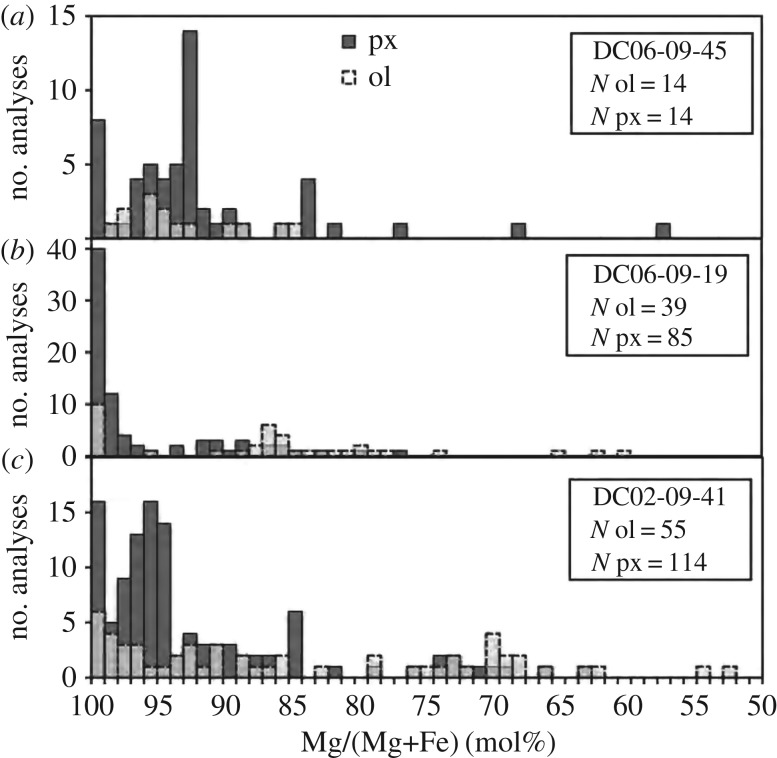
Frequency histograms showing the compositions Mg/(Fe + Mg) (mol%) of olivines (dashed line, light grey) and pyroxenes (filled line, dark grey) in crystalline assemblages of ultracarbonaceous micrometeorites (UCAMMs)(*a*, DC06-09-45; *b*–DC06-09-19 and *c*—DC02-09-41) with the number (N) of measurements made in each sample (reproduced with permission from [272], fig. 5. Copyright © 2012 Elsevier). For comparison to figure 14, Fe/(Fe+Mg) (mol%) = 100 – (Mg/(Fe+Mg)) (mol%), i.e. 100 (mol%) on scale Mg/(Mg+Fe) equals 0 (mol%) on scale Fe/(Mg+Fe).

**Figure 14.**
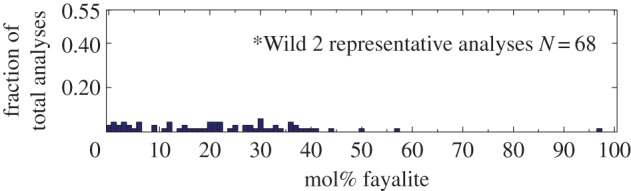
Distributions of fayalite content (mol%) for olivine in (*Stardust*) Wild 2 terminal particles and chondrite matrix. *Includes analyses from 36 particles harvested from 26 tracks; data was obtained by us [12] and [15,100,165,166,178,189,203,279–281] (reproduced with permission from [12], fig. 1. Copyright © 2014 Elsevier). Representative analyses is one analysis per particle, unless individual crystallites within a single particle differ by greater than or equal to 3 mol% fayalite. (Online version in colour.)

Olivine and pyroxene compositions reach a wide span of FeO-contents but the FeO-poor and the low-end of FeO-rich compositions dominate. Olivine average compositions (and compositional ranges) in Fa mol% =(Fe/(Fe+Mg)) are Fa7 mol% (Fa2–16), Fa13 mol% (Fa0–Fa41) and Fa16 mol% (Fa0–Fa49). We emphasize the olivine and pyroxene (crystals) with Fa ≤∼15 mol% dominate by number over more Fe-rich olivine and pyroxene as shown in figure 13. We emphasize the difference between the range of figure 13 and figure 14. The relatively Fe-poor and correspondingly relatively Mg-rich contents of the olivine in UCAMMs are quoted in [272] as Fo mol% = 100 – Fa mol%. For the smaller diameter minerals, there is a greater range of Fe-contents [272], fig. 6. With respect to figure 14, the ‘forsteritic’ olivine is Fa0–Fa5.5 (FeO<5.5), the ‘FeO-rich’ olivine is 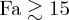 (FeO>13), and Fa5.5–15 is referred to as ‘intermediate’ [12]. ‘Wild 2 [81P] olivine does not exhibit a bi-modal distribution and is entirely lacking a forsteritic frequency peak [compared to all chondrites except CVs and Ningqiang], indicating a very different assemblage of material’ [12]. The forsteritic frequency peak also is present in the UCAMMs.

UCAMMs have significantly high abundances of carbon in highly disordered nano-structures made of nm-sized polyaromatic layers [272]; the highly disorganized layers are not without local order so it is not amorphous carbon^26^ like in some meteorites^27^ and some IDPs [112,282,283] nor is it poorly graphitized carbon as seen in other IDPs [128]. In UCAMMs, 48–88% of the surface area is carbon, which translates to carbon of 45±10 wt%, 28±6 wt%, 58±14 wt% (corresponding to the three UCAMMS shown in the silicate composition-histogram plot, from top to bottom). There also are ultracarbonaceous IDPs that look exactly like UCAMMs and probably are the same primitive matter [113]. UCAMMs have some areas with significant deuterium enrichments (δD > 5400‰ [273]) that commonly are prescribed to presolar grains. The areas with D/H >10^3^ extend over 135–280 μm^2^ and 65–200 μm^2^ in particles 19 and 119, respectively [272], fig. 3, A & C. UCAMMs have both a high carbon abundance and a pyroxene-to-olivine ratio >1, which is the same correlation between high carbon abundance and pyroxene-dominance as observed in anhydrous IDPs by [139] (§1b). The oxygen isotopes have been measured for two other fragments of UCAMMs (DC06-05-65 and DC-06-07-41) [274]: the latter has a bulk composition that is enriched in ^16^O and is approximately compatible with the ^16^O-content of CAIs [246] as well as having a highly heterogeneous distribution of δ^17^O and δ^18^O. These ^16^O-rich minerals in UCAMMs have similar to the signatures of the first CAI condensates and similarly ancient AOA particles (§4) [274]. LIME olivine, kosmochlor and carbonaceous nanoglobules in UCAMMS add to the connections between UCAMMs and the primitive cometary materials in both *Stardust* and anhydrous IDPs [284].

A different UCAMM (DC-06-05-94 or UCAMM-94) contains up to 80 vol% organic matter and appears rich in N and S. ToF-SIMS shows N-bearing species that are very common when samples contain S and C and N (in any form) (e.g. CN^−^, CH_2_N^−^, C_2_H_2_N^−^, C_2_H_2_N_2_O_2_^−^) and S-bearing species (e.g. S^−^, HS^−^, SO^−^, SO_2_^−^, SO_3_^−^, CO_2_S^−^, CO_2_HS^−^) [275]. In general, in ToF-SIMS, the largest fragments are more interesting and can give insights into the structure of the bearing molecules, provided that it is not coming from contamination. For example, part of the S in UCAMMs at least appears to come from the organic phase.

Bringing to mind the newest data on the coma of comet 67P, the *Rosetta* ROSINA mass spectrometer measured a wondrously diverse ‘zoo’ of gas-phase carbonaceous molecules including S-bearing molecules (the zoo’s skunks). The simplest molecules (H_2_S, OCS, SO, SO_2_ and CS_2_) are present in cometary ices [196–198], so there is not necessarily a genetic link to the S present in the dust [198]. However, a bit more complex organic S-bearing molecules and also reported by ROSINA [199] could be more interesting in the perspective of a comparison with organic sulfides or sulfur oxides possibly present in UCAMMs (to be verified, not given in the above list) or in the organic matter of carbonaceous chondrites.

In another UCAMM (D05IB80), a large domain of organic matter (10 μm × 20 μm) rich in N contains nitrile, imine and amide [285]. This UCAMM may have been very slightly aqueously altered because of minor phases of Ni-bearing pyrrhotite and GEMS-like materials without Fe–Ni alloys. Formation of the organic matter may have occurred via photochemical synthesis followed by cycles of warming of ice (a H_2_O–CH_3_OH–NH_3_–CO cycle [286]) [285].

In the broader context, UCAMMs have as high or higher carbon abundance compared with the prior study of carbon in anhydrous IDPs [139]. Duprat *et al.* [273] remark, ‘The exceptionally high carbon content of the UCAMMs equals or exceeds that of the most C-rich IDPs [128,139] and falls in the range of the CHON particles detected in comet 1P/Halley [289]. Both the crystalline and amorphous silicates in the UCAMMs are comparable to those detected in the dust of different comets [17]’. Recall that 25% of CHON in Halley was elemental carbon [17,114,288,289]. From the perspective IR spectroscopy of comet dust, UCAMMs have materials most similar to the comae compositions of observed comets. IR spectra are well fitted with a minimal number of components that include Mg–Fe amorphous silicates, amorphous carbon^28^ and Mg-rich crystalline olivine (forsterite) [17,20,33,41,53,144,146,148,150]. These comet dust compositions are deduced from thermal models of the IR emission of fine-grained dust (§8). Note that micrometre-sized FeS grains, which are abundant in UCAMMs, are yet to be sufficiently included in thermal models. FeS is basically not discernible via IR spectroscopy because of its lack of spectral features; FeS would be a warm dust component possibly comparable to amorphous carbon, but the lack of optical constants over a contiguous range of wavelengths from visible through to the IR contribute to its lack of use in thermal models for IR spectra of comets. Only submicrometre FeS grains would not be optically thick to themselves enough to produce the broad resonance centred at 23.5 μm that definitively has been detected in a couple of external protoplanetary discs and IDPs [291,292].

UCAMMs carbonaceous matter is most similar to one of the four textures described in *Stardust* and in IDPs: smooth (shapeless chunks with a smooth surface) [112]. A N-rich smooth-texture carbonaceous phase in UCAMMs (by XANES analyses) contains aromatic carbon, nitrile, ketone and carboxyl, imine and amide [276]. Dartois *et al.* [195] suggest, ‘this phase may have been formed and processed by irradiation of N_2_ and CH_4_-rich ices at the surfaces of large bodies [because of the need for surface water] in the outer regions of the protoplanetary disk’. The N-poor granular-texture carbonaceous phase is associated with the mineral-rich regions containing sub-micrometre Mg-rich olivines and pyroxenes, Ca-rich pyroxenes, Fe–Ni metal, Fe-sulfides and abundant GEMS. The N-poor carbonaceous phase is associated with inner ppdisc crystalline silicates so it may have formed in different environs compared with the N-rich organics [272], such as in the near-surface layers of the ppdisc as hypothesized by Ciesla & Sandford [6]. A direct comparison has been made between the organic matter in UCAMM Cluster 19 and the IOM in CR chondrites because the UCAMM has a similar composition of C/H and D/H ratio as the bulk composition of CR IOM [191].

UCAMMs probably are cometary particles. Since UCAMMs fall to the Earth, their GEMS are collected without silicon oil and their GEMS show smaller nor no element/Si depletions in contrast to GEMS in IDPs that are gathered from the stratosphere on silicone-oil-laden collectors and where silicon oil contamination is irreversible [76]. UCAMMs’ GEMS have no Si enrichments and hence no element/Si depletions and therefore cannot be argued to be solar system materials based on depleted element/Si ratios, which is an argument postulated by Keller & Messenger [81] to argue for GEMS origins in the ppdisc. The dynamical origin of UCAMMs from comets or asteroids is inconclusive, however, because UCAMMs’ entrance velocities into the earth’s atmosphere are lower compared with typical cometary IDPs. UCAMMs have fine-grained fluffy textures and appear unaltered or minimally altered by heating during atmospheric entry, because they lack vesicles and magnetite rims [273]. High S- and low Si-abundances promote the cometary origin of the GEMS in UCAMMs [68]. Thus, for UCAMMs, their textures, high carbon and low-Ca Mg-rich pyroxene and Mg-rich olivine, D/H enrichments and GEMS contribute to the view that UCAMMs represent a particular reservoir of cometary materials. UCAMMs have properties distinct from *Stardust* samples.

The *Rosetta* COSIMA instrument identifies in the coma of 67P a component of the dust that is high molecular weight organic matter [190], which is similar to the organic matter in IDPs [108] and UCAMMs [195]. Two 100 μm size regions, ‘Kennith’ and ‘Juliette’ are reported on and are representative of seven other particles. COSIMA’s mass spectra of the organic matter in Kennith and Juliette are best represented by reference spectra of IOM in carbonaceous chondrites (Orgueil and Murchison); three aspects in which they are similar in that they both have high molecular weights, lack carbon-bearing ions with *m*/*z* ratios more than 50, and have lower H/C ratios. There is no evidence for organics like carboxylic acids, aliphatics, PAHs or amino acids that are of lower molecular weights and higher H/C ratios. The organic matter in 67P is distinct from CC IOM by its higher CH_*x*_^+^/C^+^ ratio, which translates to a higher H/C ratio. A higher H/C ratio is attributed to the comet being a more primitive body than CCs because parent body processing tends to lower the H/C ratio in IOM in CCs [191]. Fray *et al.* suggest the source regions for comet 67P’s organic matter could be the ISM [192] or the cold outer ppdisc [6].

Given that we are calling out UCAMMs as cometary materials, there are potential connections between UCAMMs and chondritic materials. Let us consider the discussion of CR precursor materials summarized above (§5e) (e.g. [244,264]). The formation of type I and type II chondrules in CR chondrites calls for assemblages’ precursor materials that possess a wide range of carbon contents and different but distinct Fe-contents. Dobrica *et al.* [287] calls out UCAMM assemblages of olivine, pyroxene, sulfides, GEMS and SiO_2_-rich glass as ‘equilibrated’ because each assemblage has similar Fe-content of the silicates. Globally, UCAMMs are unequilibrated, in the same manner as seemingly all cometary materials, because there are different oxygen fugacity components (different Mg-contents for the silicates) and differing amounts of carbon (from practically none to more than 50% carbon) in close proximity and at submicrometre to micrometre scales. Cometary porous aggregates of unequilibrated materials could be some of the dustballs called for by meteoriticists. CR type I precursors also need enhanced S, presumably in sulfides [264], and S-rich GEMS also occur in UCAMMs, possibly providing this precursor material. Lastly, UCAMMs have organics similar to CR IOM [191]. UCAMMs seem to have aspects of precursor materials like high-carbon and S that are needed for CR type I chondrule-formation (§5e).

## 7. Nebular condensation

**Figure 15.**
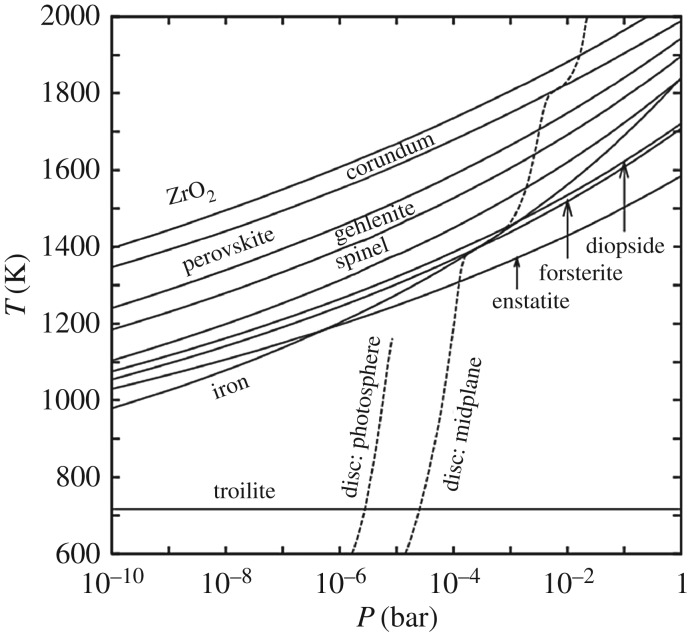
The stability limits of the important solid phases in the pressure regime of interest for accretion discs. Also shown are the typical P–T-combinations in the midplane of the disc and in the disc atmosphere. As the gas cools in the mid-plane, at *P*>10^−3^ (bar) Fe-metal grains condense out before forsterite (Mg_2_SiO_4_) and enstatite (MgSiO_3_) (reproduced with permission from [26], fig. 2. Copyright © 2004 Elsevier). See also [44].

The Fa-contents of the majority of olivine and pyroxene in UCAMMS can be produced via condensation. We briefly highlight condensation models for materials in our protoplanetary disc to provide contrast to the analyses of geochemical igneous systems. Condensation models of cooling gases in the ‘solar nebula’, i.e. our ppdisc of solar gas composition, produce forsterite and Fe metal, followed and enstatite and FeS (troilite) at much lower temperatures (figure 15) [26,44]. FeS can form by condensation processes, and also forms when primitive dust melts, and also forms during aqueous alteration. So, FeS is not usually denoted ‘primitive’ although it can be.

Condensation with partial isolation (CWPI) models [293,294] remove the elements and molecules that condense into minerals from the gas phase as condensation proceeds. CWPI models predict a preponderance of Mg-pure olivine (forsterite) and Fe metal as well as show lower pressure regimes where clino-pyroxene condenses (Cpx) [294], fig. 4. Condensation models that consider elemental enrichments in the gas via dust vaporization [294,295], i.e. dust enrichments or dust/gas ratios of 10^3^–10^4^ that no longer are solar composition, can yield Fe-rich olivine of Fa25. By vaporizing CI dust, the oxygen fugacity (log( *f*_O_2__)) increases due to the release of oxygen in silicate as well as oxygen presumed locked in minerals due to aqueous alteration. If vaporization of dust-enrichments of 10^4^ for CI-dust are followed by subsequent condensation, then olivine yields a higher FeO-content. However, dust enrichments great enough to create the higher FeO contents cannot reproduce the Na-enrichments (Na_2_O) and FeO systematics in both bulk chondrules and the (glassy) mesostasis, so type I and type II chondrules did not condense directly from nebular gases [294,295], but instead they must be at least second generation melts, probably melts of dustballs of a mixture of precursors. Fedkin *et al.* [296] show that if shock conditions are dust-rich and water-rich (dust and water enrichments by 600× and 550×, respectively, relative to solar composition) and are at 100× higher pressure (*P*_tot_≈4×10^−2^ bar) and occur for 100× shorter heating times, then a range of FeO-contents can be achieved coupled with undetectable internal isotopic heterogeneities for type II porphyritic olivine (PO) textures; examination of [296], fig. 19 shows that type II PO has a composition spike at *X*_Fa_<10 and a tail to *X*_Fa_∼33. However, no methods have been suggested yet that can produce such high dust and water enhancements in shocks.

**Figure 16.**
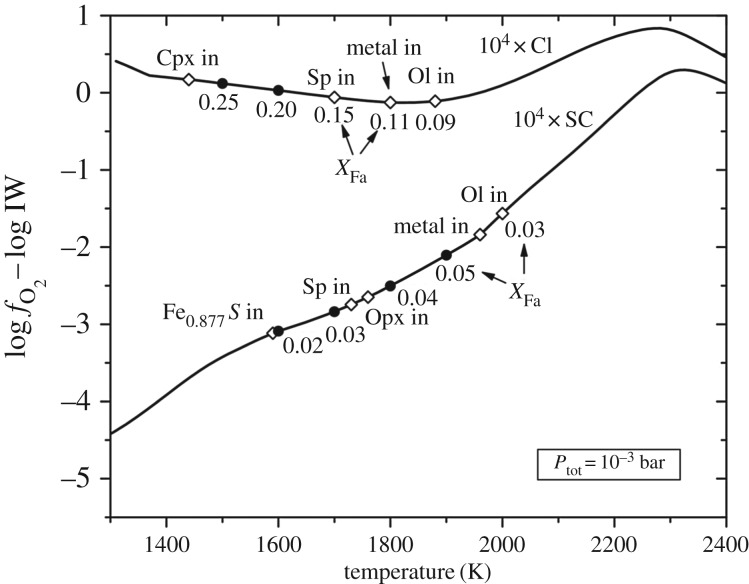
Logarithm of the oxygen fugacity relative to the iron-wüstite (IW) buffer as a function of temperature in systems enriched in CI and SC dust by a factor of 10^4^ relative to their complementary gas at *P*_tot_=10^−3^ bar. Symbols and labels are as in [297], fig. 2: open diamonds indicate the condensation temperatures of the phases; filled circles and numbers next to them are the mole fraction fayalite (*X*_Fa_) in the coexisting olivine. Both the FeO content of the liquid and *X*_Fa_ increase continuously with decreasing temperature in the CI dust-enriched system (reaching *X*_Fa_=0.25). By contrast, in the SC dust-enriched system, the FeO content of the liquid and *X*_Fa_ reach a maximum [*X*_Fa_=0.05] at 1960 K, the metal condensation temperature, and then both fall as temperature falls. Sp=spinel; Ol=olivine; Cpx=clinopyroxene; Opx=orthopyroxene (reproduced with permission from [297], fig. 3. Copyright © 2016 John Wiley & Sons, Inc.)

Fedkin & Grossman [297] have modelled condensation at *P*=10^−3^ bar with dust enrichments of 10^4^× CI-like dust and 10^4^× SC dust. In figure 16, condensation proceeds from right to left as the temperature cools for CI-dust in the upper curve: starting at 1820 K and at oxygen fugacity of log(*f*_O_2__) = IW − 0.1, olivine has *X*_Fa_=0.09 and reaches *X*_Fa_=0.25 at approximately 1500 K, that is, the olivine reaches Fa25. The precursor dust provides 99.97% of the oxygen atoms that raise the oxygen fugacity to levels where FeO-rich olivine grains condense. The FeO content of the liquid and *X*_Fa_ increase continuously with decreasing temperature in the case of CI-like enriched dust. Solar nebula condensates (SC) provide insufficient oxygen even at the same dust enrichments so the end product is *X*_Fa_=0.04. CI-like dust contains more oxygen because it is presumed to have mineral grains metamorphosed via aqueous alteration and then ejected from a water-rich parent body. Another way to state this constraint is that CI dust has 2.3 times as many oxygen atoms per 10^6^ Si atoms as SC dust.

To require CI dust as precursor material, which is needed to condense olivine with Fa=25 mol%, seems like a ‘chicken-and-egg’ problem because aqueous alteration on parent body occurs prior to condensing the dust that appears to constitute chondrite matrix grains as well as cometary grains like *Stardust* olivine grains. The chondrite matrix grains and *Stardust* olivine grains are more Fe-rich than is possible for precursor condensates (according to the models). This is one reason to think the Fe-rich olivine grains are melts of precursor dust balls rather than condensates from dust-laden gas. On the other hand, the submicrometre- to micrometre-sized olivines in UCAMMs that are dominantly (by number) Fa0–Fa25 could be considered compositionally consistent with condensates from 10^4^ CI-dust-enriched regions.

We reiterate: current condensation models cannot produce Fa36 that exists in *Stardust* ‘Iris’. The igneous particle ‘Iris’ was discussed (§2) as an example of the physical parameters derivable by modelling the crystallization of a melt. Specifically, spinel embedded within the central olivine (Fa36) and the surrounding Ca-rich pyroxene determined the cooling rate of less than or equal to 100°C h^−1^ at an oxygen fugacity of log(*f*_O_2__)=−12(IW − 0.25) and demonstrated a quench temperature (falling out of thermal equilibrium) at approximately 950°C. The Fe- and Mn-contents of the resultant mineral ensemble were determined by the concentration in the melt. A precursor dustball was composed of an aggregate of fine-grained material, perhaps with a combination of olivine condensates, Fe metal and commonly hypothesized fragments of prior generations of chondrules. Rapid-heat-zapping of dustballs can create assemblages of minerals and glass that include olivines of higher *X*_Fa_ than would be produced via condensation from nebular gases and evaporated dust. This highlights the importance of considering igneous particles as members of the cast of primitive matter in cometary dust.

## 8. IR spectroscopy of comets

Let us consider what we know about cometary comae dust from fitting thermal models to mid- to far-IR spectra of cometary comae.^29^ IR spectra are best-fitted by a few materials that include amorphous silicates, amorphous carbon and Mg-rich crystals with crystal mass fractions ranging from 20 to 75%.^30^ An example of a high value of the silicate crystal mass fraction is *f*_crystal_=0.75 for comet Hale–Bopp at perihelion. Compared to perihelion at 0.93 AU, at 2.8 AU Hale–Bopp had a lower contrast silicate feature and a smaller crystal mass fraction of *f*_crystal_≃0.5, with different modellers deriving similar values of *f*_crystal_ of {0.60, 0.50, 0.44} for, respectively, {[35,162,300]}. Examples of low values of the silicate crystal mass fraction are 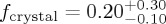 for Oort cloud comet C/2012 K1 (Pan-STARRS) [149] and *f*_crystal_=0.14±0.04 for Oort cloud comet C/2007 N3 (Lulin) [148]; comet Lulin’s grain size distribution has a steep slope (*N*=4.2) and peaks at grain radius 0.9 μm as well as having moderate porosity particles (fractal porosity parameter *D*=2.73 [41]). For a partial compilation of crystal mass fractions, see [149]. The crystal mass fractions deduced from thermal models of IR spectra are similar to laboratory examinations of UCAMMs where *f*_crystal_∼25%. When crystal resonances are present in IR absorption spectra of anhydrous IDPs, the crystal mass fraction are 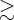
20% [79] (J Bradley 2008, personal communication). The range of crystal mass fractions in anhydrous IDPs totally depends on the IDP: some are almost all GEMS, some are almost all crystals, some are almost all organics, and there are mixtures of these materials. Recall that remote sensing is a sampling of the coma. In comparison, laboratory investigations are on samples of very tiny masses of material.

There does not yet appear to be a clear distinction in crystal mass fractions between Oort cloud versus Jupiter Family comets; this is in agreement with predictions of the ‘Nice model’ for comets arising from the trans-Neptune region and the two dynamical families arising from different orbital excitation mechanisms (cf. [301]). We are still dealing with small number statistics with only a couple of dozen high signal-to-noise IR spectra spanning around 7.5–35 μm ; JWST holds promise to contribute more high fidelity IR spectra to ascertaining whether there are statistically significant differences between the comae dust properties of the two dynamical families [302].

**Figure 17.**
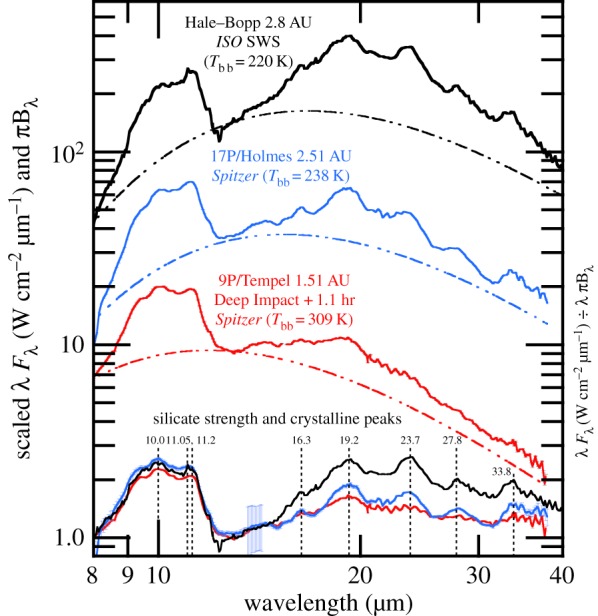
Three comets with high-contrast silicate features and with prominent forsterite (Fo100) resonant peaks designed by wavelength. Shown are the *ISO* SWS spectrum of Oort cloud Hale–Bopp [32,41,162,150], and the *Spitzer* IRS spectra of Jupiter Family comets 9P/Tempel 1 at +1 h post-Deep Impact [35] and the 17P/Holmes post-outburst [303] . (Online version in colour.)

**Figure 18.**
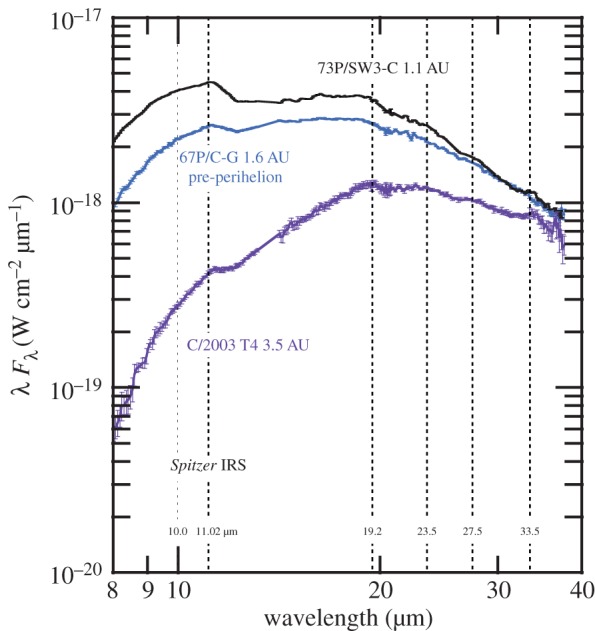
Three comets with low-contrast silicate features and with weak forsterite (Fo100) resonant peaks. Their grain size distributions have grains as small as around 1 μm radius. Shown are the *Spitzer* IRS spectra of 73P/Schwachmann-Wachmann 3°C[304], as well as (MSP Kelley 2016, personal communication) 67P [305] and C/2003 T4 [306]. (Online version in colour.)

**Figure 19.**
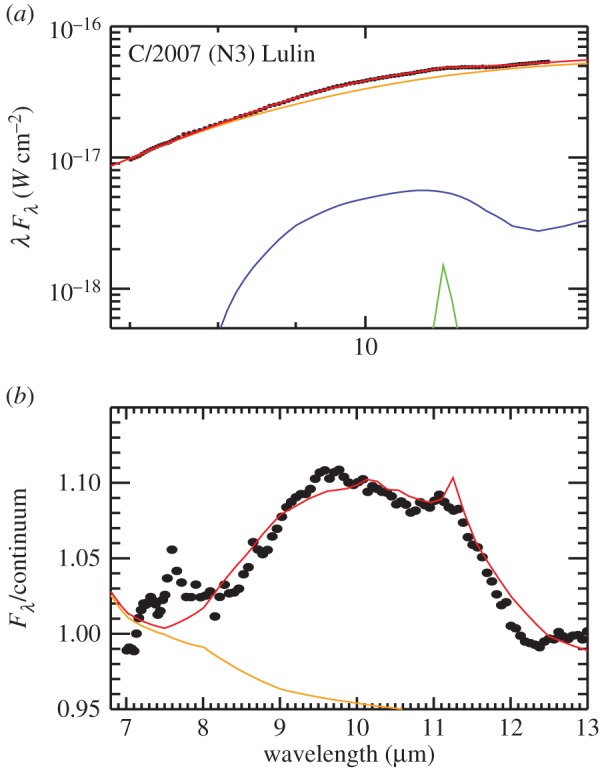
(*a*) *Spitzer* IR spectral energy distribution (SED, log(λ*F*_λ_) versus log(λ)) of comet C/2007 N3 (Lulin) with weak spectral features, which indicates its coma has micrometre-sized grains and low porosity aggregates. (*b*) Silicate feature strength (*F*_λ_/continuum). Comet Lulin has a very weak silicate feature and compact porous particles; polarization values also reveal compact porous aggregates [148] (reproduced with permission from [148], fig. 8. Copyright © AAS). (Online version in colour.)

The fine-grained forsterite (submicrometre to micrometre radii crystals) are the type of mineral expected from condensation from the solar nebula [17] (§7). Figure 17 shows the three comets with the highest contrast spectral peaks from crystalline silicates by which we can best identify and model comae dust compositions. The vertical lines show the wavelengths for forsterite (olivine with *X*_Fa_=0). All three comets have forsterite peaks with similar central wavelengths, feature asymmetries and relative intensities, i.e. they have high Fo crystal mass fractions (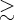
50%) and similarly shaped forsterite crystals best-fitted with ‘equant’ rectangular prisms [155]. Forsterite crystal shape is key to best-fitting the IR resonances; ellipsoidal shapes have resonances at too short a wavelength to best-match the observed crystal resonance wavelengths. Each of the three comets in figure 17 has a high feature contrast (approx. 3 in figure 17, bottom) or large ‘silicate band strength’^31^ because their comae grain-size distributions extend down to submicrometre-radii. By contrast, three comets are shown in figure 18 have moderate silicate feature strengths (approx. 1.2) that are attributable to their comae grain size distributions having grains only as small as approximately 1 μm. Comet Lulin, discussed above, has a weak silicate feature strength (approx. 1.1) (figure 19).

Only Hale–Bopp when close to perihelion revealed strong distinct resonances from forsterite *and ortho-pyroxene* [150], and some other comets under analyses are revealing ortho-pyroxene [299]. The wavelength positions of the distinct spectral peaks for forsterite can match laboratory spectra of olivine with *X*_Fa_=0–10 [154], fig. 2, but cannot be fitted with Fe-rich olivine. The 11.15–11.2 μm Fo-peak is expected to shift to longer wavelengths by +0.15 to +0.25 μm for Fa30–Fa40, and this shift is not discerned in the existing *Spitzer* spectral dataset on comets with its high signal-to-noise ratio spectra (error bars are shown in figures 17 and 18). *Spitzer* IRS spectra cover the far-IR resonances of olivine and the far-IR resonances have larger shifts to longer wavelengths for Fe-rich olivine, so the full spectral coverage of *Spitzer* is key to ascertaining the presence or setting limits on the Fe-content of the olivine, i.e. the crystalline silicates. The far-IR resonances also can be measured by *SOFIA* and *JWST*.

T Tauri discs, external analogues for our protoplanetary disc (introduced in §2), also have IR spectra with crystalline silicate features dominated by Mg-rich crystalline silicates, i.e. forsterite with some enstatite [216,308] . The crystalline mass fractions of external protoplanetary discs are approximately 10%, which is lower than what we deduce for comets from thermal models to IR spectra.

We note that models for cometary polarization properties are best accomplished with a combination of solid grains and porous particles [309], which parallels IR thermal models. Visible and near-IR polarization models employ a mixture of optical constants for silicates, absorbing carbon [309,310], as well as refractory organics that mimic Halley-like CHON materials [311,312].

Of all cometary extraterrestrial samples and *in situ* measurements, the compositions of these three comets are more similar to Halley and to the fine-grained components of UCAMMs than to the full suite of *Stardust* materials that include the olivine matrix grains discussed here in detail. *Stardust* does contain forsterite crystals as terminal particles but they do not dominate by number; rather, *Stardust* has a flat frequency distribution of *X*_Fa_ for the olivine [12,179].

## 9. Commentary

Let us assume that our knowledge of geochemistry is fully capable (not lacking in any capability) of explaining *Stardust* olivine as independent igneous systems. Then the higher Fa mol% of olivine matrix grains in *Stardust* and in type II chondrules in chondrites is verifying the concept that chondrule precursor materials probably are composed of melt-generated-olivine, i.e. heat-zapped dustballs. However, FeO-rich olivine is not spectrally discernible in IR spectra of cometary comae from *Spitzer* that has the full wavelength coverage to sample the resonance features near 11.27–11.4 μm as well as in the 22–25 μm region and at high signal-to-noise ratio. If we assume all comets have similar compositions as *Stardust* samples and the giant CP IDPs recently reported on, then the FeO-rich olivine that is assumed to be present in comet dust distributions is (somehow) not seen in the IR spectra. If we assume that all comets do not have the same range of FeO-contents in their crystalline silicate dust, we still have the conundrum that we have yet to unequivocally identify olivine with *X*_Fa_ > 10 mol% in IR spectra.

Alternatively, we might think that *Stardust*-like cometary materials are a rarity. However, giant CP IDPs under current study show the same incredibly broad range of Fe-contents and Mn-contents as *Stardust* [12,178,179]. So, *Stardust* is not unique. Moreover, preliminary reports of COSIMA measurements of about a dozen refractory grains suggests that comet 67P may have high Fe-contents for the silicates or FeS is with silicates in the COSIMA beam (§1) [16].

Fe-rich olivine has a higher IR absorptivity than Mg-rich olivine, so if the sizes of Fe-rich olivine were large enough to make the particles optically thick to themselves, perhaps Fe-rich olivine could be hidden from spectroscopic detection. Preliminary computations of fayalite with rectangular-shaped crystals using the DDSCAT code, similar to the techniques in [155], suggest the Fe-rich olivine would need to be larger than approximately 5 μm for opacity effects to start to significantly affect the far-IR resonances. Fe-rich olivine in *Stardust* are 5–30 μm-size but these same composition crystals are not that large in giant CP IDPs. So, a large size for Fe-rich olivine is not the simple answer. The discussion presented here strongly motivates solving the puzzle of why the Fe-rich crystals are absent from IR spectra of comets.

One may ask: Do we just have enough comet spectra to make a proper statistical assessment? Can we significantly expand on the number IR spectra of comets? *JWST*’s IR capabilities and sensitivity will allow the study of many comets at larger heliocentric distances. However, at larger heliocentric distances the comets are less active, and Hale–Bopp had much less crystalline material at 2.8 AU compared with at 0.93 AU [41,150,162]. This means that we must still use ground-based telescopes and SOFIA to assess the dust composition of comets that are bright and productive at smaller heliocentric distances (approx. 1–3 AU).

Note from the perspective of a comet dust observer and analyst: the possibility of incorporation of cometary materials is called out as probably not part of the chondrule-formation story as told by meteoriticists [82], partly because the evidence of primitive cometary material is easily obliterated and without hard evidence the connection is not testable. We note that the regimes of high oxygen fugacity needed to form type II olivine are sought after by enhancing the dust/gas ratio by 10^4^ or by bringing in water ice (100–500× for CR type II chondrites). GEMS are abundant in some cometary samples and GEMS transform to Fe-rich olivine when heated at greater than or equal to 900°C, so cometary materials could be a source of precursor materials for chondrule formation. The mere size difference between submicrometre-sized GEMS and 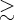
10 μm sized Fe-rich olivine grains have been one argument against this idea. Shocks may compact aggregates and in fact this is required to have the correct opacity for the fast cooling rates needed for chondrule-formation in shocks [254]. Also, some cometary dust collections like UCAMMs have abundant carbon, which is suggested to be a critical reducing agent to drive the oxygen fugacity down in regions of type I chondrule formation. We suggest that cometary primitive materials be not only considered to be possible products of chondrule-formation but also considered as contributors to the precursor reservoir of type II chondrule-formation. The diversity of primitive matter in comets is considerable but the diversity is not uniform across all cometary dust collections/samples.

In conclusion, we ask: Where in cometary IR spectra are the larger (5–30 μm) single Fe-rich crystals that are the focus of *Stardust* discussions and chondrite type II olivine matrix grains? Is comet 81P’s late formation a rarity or the norm?

## Supplementary Material

SUPPL. Chondrites, Chondrules, Complimentarity and The Depletion Pattern

## Footnotes

1 Strictly speaking, ‘Mg–Fe amorphous silicates’ may be analogous to but are not necessarily the same material as ‘GEMS’, which are cometary primitive matter based on laboratory examinations. The ISM shows absorption features are well-matched by Mg–Fe amorphous silicates (glass) [21] made in the laboratory by rapid cooling of a melt (B Begemann 1996, personal communication). Cometary primitive matter includes GEMS, which well-match the ISM absorption features [22,23]. GEMS are Mg-rich amorphous silicate matrices with iron (nickel) and sulfides [24]. GEMS are not the same material as Mg–Fe amorphous silicates, although GEMS are the best candidates for ISM ‘Mg–Fe amorphous silicates’. We speak of GEMS when we are strictly talking about samples of cometary materials. When referring to inferred properties of ISM silicates or modelled properties of cometary comae dust from IR spectra, we say ‘Mg–Fe amorphous silicates’.

2 Amorphous silicate means amorphous-type [17], glassy, non-crystalline. Amorphous silicates may have non-stoichiometric compositions [48]. Optical constants exist for Mg–Fe amorphous olivine and amorphous pyroxene [21,49] that were made by, respectively, rapid cooling of melts and the sol–gel method.

3 Minerals are crystals, so olivine is a crystal and pyroxene is a crystal. A material near the composition of olivine and with an amorphous (glassy) texture is denoted amorphous olivine.

4 Note that there are other amorphous materials in CP IDPs that appear unrelated to GEMS [69].

5 Presolar signatures are identified typically in the most robust dust species including silicates [65,72], SiC and TiC, corundum, spinel, hibonite and oxides [73,74].

6 When TEM mapping, there definitely are widely varying levels of silicone oil contamination that depend on the particle, who did the hexane washing and when, the non-uniform effects of electron-beam irradiation, as well as the silicone oil having mobility. Efforts are being made to calibrate near-GEMS regions of IDPs with Si-XANES using spot sizes similar to the size of the GEMS grains [89]. However, this report is challenged by (i) low number statistics—this is one ∼0.4×0.5 μm GEMS grain, well isolated from other grains and more easily washed clean of silicone oil, so more need to be done and with controls; (ii) silicone oil was not subjected to the same e-beam conditions as the particle, and e-beam exposure can account for edge energy shifts; (iii) there are two overlapping Si-XANES peak positions for silicone oil depending on the C content, 1845 and 1847 eV, one of which overlaps the silica aerogel position, and the low-energy shoulder at some locations is consistent with silicone oil [90] but not with forsterite (as claimed by Flynn *et al*. [89]). Clearly, this is an area of ongoing work.

7 To be specific about the compositions of the crystalline silicates olivine and pyroxene, we use the notation keyed off the mineral group. The olivine mineral group spans from Mg_2_SiO_4_ to Fe_2_SiO_4_, from forsterite (Fo) to fayalite (Fa). Notation for forsterite can be Fo100 and equivalently Fa0. Conversely, notation for fayalite can be Fo0 and Fa100. An olivine (crystal) of composition (Mg_0.7_,Fe_0.3_)_2_SiO_4_ can be denoted Fo70 or Fa30. Using parallel reasoning, a pyroxene crystal of composition MgSiO_3_ is Enstatite or En100.

8 The point-count analyses [79] give equal weight per volume, so what they provide, if you average them all together, is volume-averaged composition, or bulk composition. The bulk composition is the same as what you would derive if you put a big spot on the entire particle section *en masse*. For the point-count analyses, a large olivine/pyroxene crystal is weighted more than a small one. Although crystal identity was not confirmed by diffraction, the crystalline phase was based on imaging and a pyroxene composition was based on the Mg/Si=1, i.e. which is its corresponding position in the ternary diagram. If the *Stardust* compositional analyses of olivine are limited to 1/crystal, then they do not reflect the volume-averaged composition. That is, they over-weight small crystals and under-weight large crystals in their contribution to a bulk composition of *Stardust* olivine. Hypothetically, if large crystals in *Stardust* tend to be more Mg-rich and smaller ones tend to be more Fe-rich, then the histogram by Frank *et al*. [12] will indicate more Fe content than the actual bulk composition of *Stardust* olivine. Both types of data are important, which is why we are explaining clearly what they represent. Both approaches are presented in the literature. For example, consider trying to understand the complementarity between chondrules and matrix: knowing the average composition of each and the volume fraction is important (electronic supplementary material, section (a)).

9 Organic species with limited coma lifetimes have been denoted semi-volatile or semi-refractory [17].

10 Nakamura-Messenger *et al.*[107,165,166] recently created a category they call ‘hybrid IDPs’ that contain 10% phyllosilicates (smectite) in otherwise anhydrous CP IDPs with a high bulk carbon contents of approximately 3–6×CI chondrites, which is a similarly high carbon content as reported by Thomas *et al.*[139]. In prior work, an IDP with any amount of hydrated minerals would have been called a hydrous IDP [95]. Aqueous alteration experiments performed to assess the formation of phyllosilicates only produced smectite from enstatite by exposure to a pH 12 solution for 12 h at 120°C, GEMS were modified much more easily, and olivine was not affected [107]. Aqueous alteration occurs more readily for Fe-rich olivine, which was not part of the experiment.

11 Type II chondrule olivine grains, along with the analogous *Stardust* Fe-rich olivine grains, are considered second generation, or perhaps *n*th generation with *n*>2–3, because it is a melt (§2) of precursor dust and the precursor dust is the earlier (presumed first) generation dust.

12 Mechanisms for the transient heating events required for chondrule-formation have been discussed for decades and as yet there is no consensus, although the nebular shock wave model is a currently favoured scenario, because it predicts the cooling rates that in experiments produce the observed porphyritic textures [180].

13 In the discussion, the characterization of *Stardust* grains has overtaken that of anhydrous IDPs, so now it is necessary for the latter to catch up. There are virtually no minor element measurements of olivine and pyroxene in anhydrous IDPs, with the exception of the new work of Brownlee & Joswiak [179].

14 Owing to the aerogel collection bias, the survival of carbonaceous matter in *Stardust* samples is strongly debated.

15 There are some subtleties of ToF-SIMS that deserve mentioning: The matrix effect is very important in TOF-SIMS analyses and direct comparisons are rarely adequate. Analyses of more of the COSIMA data may lead to a better understanding the structure of the carbonaceous matter found in 67P dust.

16 We choose to adopt the *X*_Fa_ notation herein because of its frequent use in the chondrite literature. We note that other articles on comets may use *X*_Fo_ , as shown in some of the cited figures.

17 Fe also can be trivalent (as Fe^3+^), as in phyllosilicates.

18 The pressure of chondrule-formation is pretty unconstrained, and probably was lower than 1 bar.

19 STXM and Fe-XANES is synchrotron-based X-ray scanning microscopy and Fe X-ray absorption near-edge spectroscopy.

20 Deceleration of unequilibrated, undifferentiated comet dust from around 6 km s^−1^ to standstill in silica (SiO_*x*_) aerogel resulted in ablation, melting, sometimes abrasion at lower speeds near the ends of tracks, and condensation and quenching of the resultant intermixture. Ishii *et al.*[78] demonstrated that nanometre Fe and FeS beads similar to those typically found in GEMS are a result of this process so that impact mixing of silicates and sulfides in an incident particle can produce GEMS-like objects, like the material examined next to ‘Febo’ [208]. We do not dispute that *Stardust* must have captured a lot of fine-grained material as evidenced by the craters in Al foil, some of it tiny crystals seen by TEM, and some fine-grained material may have been GEMS.

21 The notation for O-isotopic compositions are δ^17^O, δ^18^O and Δ^17^O, which are defined with respect to Standard Mean Ocean Water (SMOW). δ^17^O (‰) ×1000=((^17^O/^16^O)/(^17^O/^16^O)_SMOW_−1), and similarly for δ^18^O. Δ^17^O=δ^17^−0.52×δ^17^O. Ogliore *et al.*[211].

22 On a three-isotope plot of δ^17^O versus δ^18^O, the relation expected from the process of preferential dissociation of the heavy oxygen in CO is called the Young & Russell line [243], which is well separated from the terrestrial fractionation line (TFL) and somewhat close to the carbonaceous chondrite anhydrous mineral (CCAM) line.

23 The ‘X-wind model’ does not work for chondrules because there is no explanation for the chondrule-matrix complementarity; the X-wind model also has challenges for CAIs. The launch zone is close to the Sun (0.1 AU) where silicates are expected to evaporate and thus silicates and type II chondrules would not survive to be expelled to large distances [260].

24 In the literature, UCAMMS also are referred to as chondritic porous micrometeorites CP MMs from Antarctica.

25 FeS is a common mineral in cometary materials. Note, FeS is not commonly considered ‘primitive’ because FeS can form by condensation (§7) or by metamorphism [277].

26 There are several types of amorphous carbons, and some are organic and some inorganic. None are minerals because they are amorphous (§1a(v)).

27 Amorphous carbon is found in IDPS, the Bells, Tagish Lake, Murchison, and Orgueil meteorites and *Stardust* particles [282].

28 The optical properties of amorphous carbon are similar to glassy carbon [41], and to graphitic carbon and pyrolysed cellulose [290].

29 The thermal models follow the pioneering work of Martha Hanner [298], whereby IR spectral energy distributions are fitted to models of flux densities of an optically thin comae of an ensemble of dust particles characterized by mineralogy and by a grain size distribution. Each grain’s radiative equilibrium temperature is computed based on its IR absorptivity Q_λ_ and the balance between absorbed sunlight and emitted thermal radiation, where Q_λ_ is computed using optical constants.

30 The silicate crystal mass fraction is defined *f*_crystal_=*m*_crystal_/(*m*_crystal_+*m*_amorphous silicate_), where m_crystal_ is from forsterite or forsterite plus enstatite (cf. [41,146,148,156,299]).

31 Silicate band ‘strength’ is defined as the flux density divided by a scaled blackbody [307].

## Data accessibility

NASA ADS has ‘On-line Data’ links for *Spitzer* IRSA archive data in figure 18 for [305,306].
