# Supplementary material for: Cometary dust: the diversity of primitive refractory grains
Source: Philos Trans A Math Phys Eng Sci. 2017 May 29;375(2097):20160260. doi: 10.1098/rsta.2016.0260 (PMC5454228; doi:10.1098/rsta.2016.0260)
Supplement: SUPPL. Chondrites, Chondrules, Complimentarity and The Depletion Pattern [file rsta20160260supp1.pdf]

1. Supplemental

(a) Chondrule Types

There are three groups of least-altered chondrites (1; 2): Unequilibrated Ordinary Chodrites (UOC), which are the most common meteorites to fall to Earth; Carbonaceous Chondrites (CC), which have a small amounts of carbon; Enstatite Chondrites (EC), which are the most highly reduced (having the least amount of iron in FeO) and are dominated by pyroxene rather than olivine. These large chondrite groups contain distinguishable chondrite classes: UOC=H, L, LL; CC=CO, CH, CM, CR, CV-reduced, CV-oxidized, CB, and CK; E=EH, EL. Acfer 094 is an ungrouped chondrite with abundant Mg-rich crystals (3), and with an affinity to CO chondrites.

Chondrites are composed mainly of chondrules and matrix. Chondrite groups and classes are distinguished clearly by chondrule composition and size (2). Using the Al-Mg and Pb-Pb chronometers (4), roughly speaking the span of ages for UOCs are older (0–3 Myr) but with some overlap with CCs exemplified by CO3 at 1–3.5 Myr (Yamato 81020) and CR2 at 1.5–3 Myr (Acfer 059). Older means having formed earlier in the evolution of the ppdisk. Note that solar composition is well-matched by the composition of the now chondrule-free 'CI chondrites' so CI- and solar- composition are often used interchangeably and elemental compositions are shown as ratios to CI.

Chondrules are mm-size, with some up to golf-ball-size ($\sim$4 cm-diam.) (5). Chondrules were crystalized from melts. Chondrule precursor materials are diverse and likely included in varying proportions: CAIs, AOAs, Mg-rich relict grains and (slightly Fe-rich) "dusty" olivines (§ 5(e)), as well as prior generations of chondrules, which is shown nicely in a sketch (Fig. 1, (4, Fig. 4)).

Chondrules are categorized as type I or type II based on whether their silicates have Fe/(Fe+Mg) < 10% or >10%, with the general value of 10% being a bit arbitrary (1). Type II chondrules dominate by number in UOCs whereas type I chondrules dominate in CCs (6). In type I chondrules, Fe is in metal and expulsion of metal blebs may have occurred during chondrule melting. The general agreement of major elements compositions in type I chondrules is shown in the Fe/CI *versus* Mg/CI plot in Fig. 3 (2, Fig. 4a). Type I chondrules have fairly uniform compositions in the major elements of Mg, Fe, Si so the reservoirs that formed type I chondrules were fairly uniform in these parameters (2). In contrast, type II chondrules have FeO-rich silicates, little or no Fe-metal, and have a higher abundance of volatile elements than type I chondrules.

Typically, chondrule compositions show a break in behavior between low- and high-Fe creating a natural dividing line. Frank *et. al.* (2014) define Fe-rich as X_{Fa}>13 *mol.%*. Schrader *et. al.* (2015) define Fe-rich as X_{Fa}>10 *mol.%*. Fe-rich olivine can mean (Fa10–Fa25) or (Fa25–Fa100), i.e., moderately or very FeO-rich. From a (§ 7), (7) show $X_{Fa} \leq 25$ *mol.%* can be produced by condensation in regions with high CI-dust/gas ratios, where CI-like dust has 2.3 times as many oxygen atoms per 10^6 Si atoms as solar nebula condensates (SC-dust). Olivine with $X_{Fa} \leq 25$ *mol.%* also can form in water-rich shocks (8). In contrast, X_{Fa}>25 *mol.%* seems achievable only by melting of dustballs of similarly high Fe-content, melting in a gas with high oxygen fugacity, and/or melting with FeO addition from the gas.

Chondrule minerals are categorized by their compositions and textures (1; 2). In decreasing order of abundance, chondrules are porphyritic olivine-pyroxene (POP), porphyritic olivine (PO), and porphyritic pyroxene (PP). Fe-poor type I chondrules of PO, POP, PP textures are type IA, IAB, IB chondrules, respectively. Fe-rich type II chondrules of PO, POP, and PP textures are type IIA, IIAB, and IIB chondrules, respectively. Minor chondrule compositions/textures include granular (G), barred olivine (BO), radial pyroxene (RP), and cryptocrystalline. Chondrule textures generally result from differing degrees of melting and subsequent cooling rates, although this is complicated by the fact that chondrules generally have witnessed multiple episodes of melting and partial evaporation.

Type I chondrules typically are POP (type IAB), having olivine in their centers and low-Ca pyroxene towards their edges that probably is to progressive condensation of SiO_2 from the gas during cooling (1; 9). In carbonaceous chondrites, type I chondrules may gain SiO_2 3–15 *wt.%*

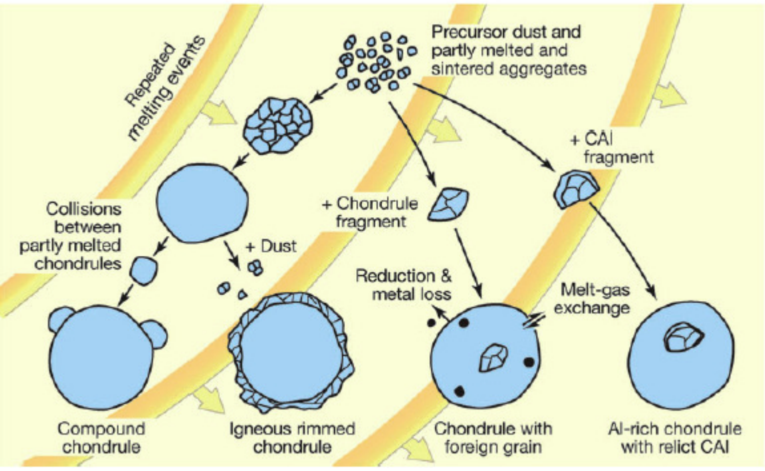

Figure 1: Sketches illustrating the processes involved in chondrule formation: heating and melting of dust, growth by collisions between solid particles and melted or partly melted objects, and exchange between gas and melt. These processes account for the existence of fragments of chondrules and CAIs within chondrules, igneous rims, and adhering chondrules on the exterior, and the growth of pyroxene around type I chondrules. [Adopted from (4, Fig. 4).]

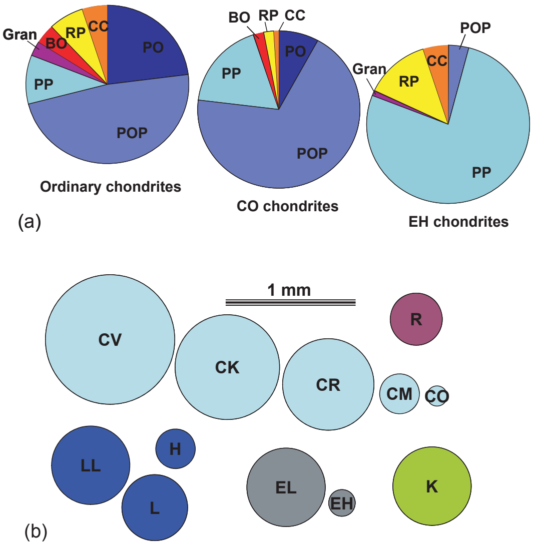

Figure 2: Properties of chondrules in different chondrite groups, based on data compiled by Scott and Krot (2003). a) Distribution of chondrule textural types in ordinary, CO, and EH chondrites. Porphyritic types include porphyritic olivine (PO), porphyritic olivine / pyroxene (POP), porphyritic pyroxene (PP), and granular (Gran). "Melted" textural types include radial pyroxene (RP), barred olivine (BO), and cryptocrystalline. b) Mean chondrule sizes of the twelve chondrite groups in which chondrules are considered to have formed in the nebula, within about 3 Myr of CAI formation. (The other chondrite groups are CI chondrites, which do not contain chondrules, and metal-rich groups CH and CB, which include materials that are suggested to have formed in a later impact plume.) [Adopted from (2, Fig. 1).]

from the surrounding gas after they were molten in an open system behavior, which probably is linked to the oxygen fugacity during the later stages of chondrule formation; thus, type I POP may be probing both precursor materials as well as the abundance/mobility of gas-phase molecules (SiO_2) during chondrule-formation (10). In discussing the Fe–Mn relation, investigators focus

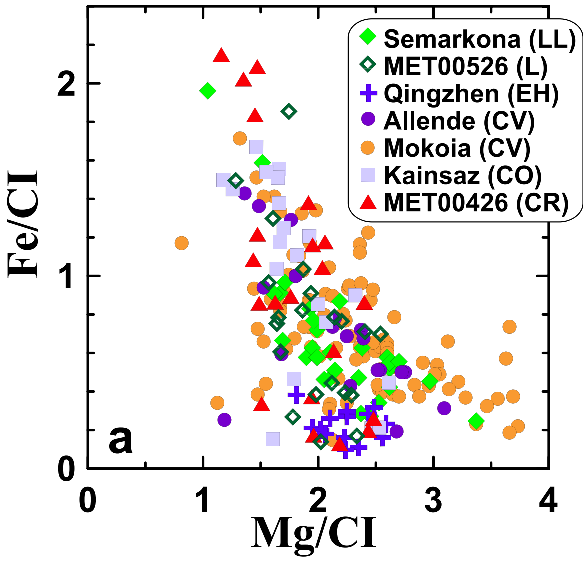

Figure 3: Bulk compositions of chondrules in primitive chondrites: Semarkona (Grossman and Wasson 1983); Allende (Rubin and Wasson 1987); Mokoia (Jones and Schilk 2009); Qingzhen (Grossman et al. 1985); Kainsaz (Berlin 2009) and Meteorite Hills 00426 (Berlin 2009). [Adopted from (2, Fig. 4); see article for references for each chondrite.]

on PO (*olivine*) and not POP, presumably to avoid the additional complications of the evolving mineralogy and oxygen fugacity during the chondrule-forming event.

Type II chondrules of PO texture (type IIA) are the focus of studies of the Fe–Mn relation and are discussed here. Fe and Mn are found together in type IIA chondrules and studies of the Fe–Mn relation is one important probe of type II chondrule formation (11). The 20 μm-sized *Stardust* igneous particle Iris is a probably a type IIA chondrule fragment (based on its small size that overlaps with microchondrules), and whose formation was modeled under thermodynamic equilibrium conditions (§ 2). To form Fe-rich silicates requires a high oxygen fugacity and a high oxygen fugacity can be attributed to high dust-particle enrichments. High dust/gas ratios coupled with the increased brittleness of FeO-rich olivine compared to FeO-poor olivine supports the concept that Fe-rich olivine matrix grains are type II chondrule fragments. In CR chondrites, Fe-rich matrix minerals, type II chondrule fragments and type II chondrules are compositionally indistinguishable (12). In the literature, type II chondrules/fragments/matrix grains they are discussed often without distinction to their shape and size. If Iris is a fragment, then its composition may not represent the composition of the whole chondrule (13). On the other hand, even though Iris is small for a chondrule, the whole particle may have been an isolated igneous event (12). Consider the possibility that *Stardust* chondrules Iris, Callie and Torajiro are examples of small and isolated igneous systems. If the smaller type II chondrules (commonly referred to as chondrule fragments) could have formed at their current size and by similar processes, then a size sorting process, possibly attributable to aerodynamics (or unspecified methods) coupled with the fact that size distinguishes most type I from type II chondrules, may point to the importance of small type II olivines as individual igneous systems in themselves. Isolated igneous systems is one explanation for the wide region hosted by CR chondrites in the Fe–Mn diagram (12; 14).

(b) Chondrule–Matrix Complementarity

The complementarity between chondrules and matrix is shown for the silicates (lithophiles) in carbonaceous chondrite classes CR, CO, CV and CM in Fig. 4 (15, Fig. 4) who used data from (16); the bulk compositions of carbonaceous chondrites are contained within the gray circle where bulk

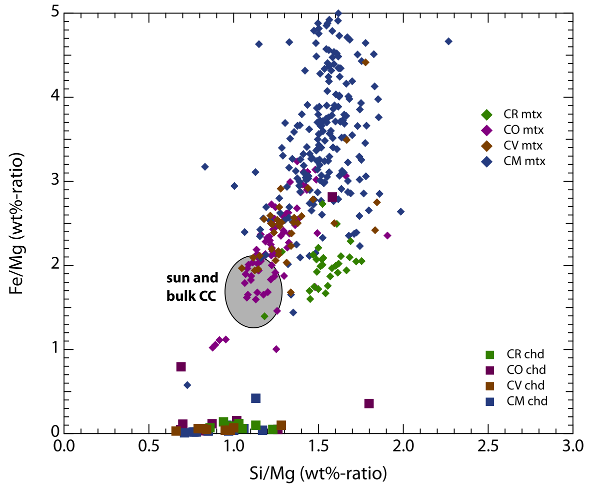

Figure 4: [Si/Mg *versus* Fe/Mg ratios for CC matrix and chondrules.] Same diagram as (15, Fig. 3) with data on matrices and chondrules of CR, CO, CV and CM chondrites from Hezel and Palme(2010). Most matrix analyses have higher Fe/Mg and Si/Mg ratios than bulk carbonaceous chondrites. The corresponding chondrule analyses are lower in both ratios. The comparatively low Fe/Mg ratios of matrix of CR-chondrites (Renazzo) is the result of avoiding metals when analyzing matrix. [Adopted from (15, Fig. 4).]

chondrite Fe/Mg declines in this order: CI=solar, CO, CM, CR and CV, *cf.*, (15, Fig. 3). The bulk composition lays between the bulk composition of the matrix and chondrules because chondrules and matrix dominate the bulk composition (6). Palme *et. al.* (2015) (15) says of CCs, "Several hundred chondrules with individual masses in the milligram range and of variable chemical compositions produce chondrites with chemical compositions constant at the gram scale (17; 18)."

Complementarity can be explained by either ('model a' in §1(c)) transfer of material from type I chondrule precursors via evaporation followed by part or full recondensation into matrix, or ('model b' in §1(c)) type I chondrules and matrix are different fractions of a single bulk reservoir. These two models are argued for in the literature and an indisputable discrimination between the models has yet to be demonstrated.

(c) The Depletion Pattern

Important subtle differences in bulk element/CI ratios exist and distinguish chondrite classes (6; 19; 20; 21; 22). Figure 5 (6) shows the depletion pattern of elemental abundance/CI for the bulk (*heavy line*) and for the matrix (*light line with points*) as a function of decreasing condensation temperature and increasing volatility. Chondrule bulk compositions are not shown but they are lower than the bulk line because enrichments in matrix over bulk are "mirrored by concomitant depletions in chondrules" (21). Bulk chondrule compositions are measured over large enough volumes of the chondrule so as to sample the bulk and are not be strongly affected by zoning or overgrowth patterns (§ 5(d)).

All chondrites have experienced some degree of parent-body aqueous alteration and/or thermal alteration, with type 3.0 being the least metamorphosed, type 1 being the most aqueously altered and type 6 being the most thermally altered. Superimposed on these trends are mineralogical and chemical changes due to impact shock. The least altered chondrites are best for studying the depletion pattern. The survival of presolar silicates and amorphous matter GEMS-like in matrix are keys to defining the least-altered chondrites. Matrix is enriched in volatile and moderately volatile elements compared to bulk chondrites. Matrix, however, is not CI composition (except in CI chondrites!) so matrix experienced some processing prior to chondrule-forming events and incorporation into chondrites.

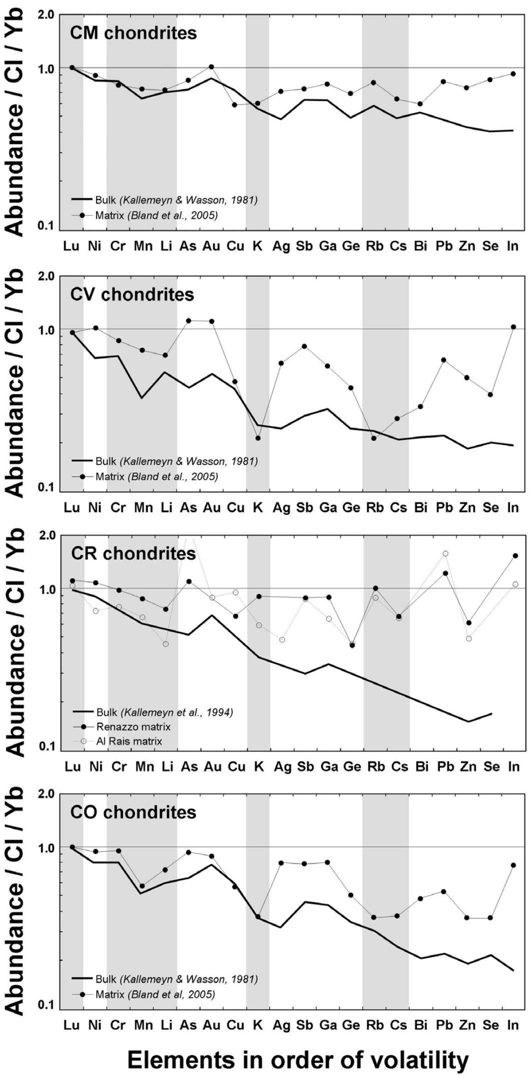

Figure 5: Average trace elements abundances for the matrices of CM, CV, CR, and CO chondrites are presented, normalized to CI chondrites and to the refractory element ytterbium [Yb] (the choice of refractory element for normalization does not significantly affect the abundance patterns). The elements are arranged in order of increasing volatility from left to right. Also shown are the average compositions of the bulk meteorites. The gray bands on the diagrams indicate the lithophile elements. Data are from Bland et al. (2005). [Adopted from (6, Fig. 5).]

Minor deviations in the bulk compositions with respect to a monotonic depletion pattern corresond to major deviations in the matrix (21). Bulk chondrule compositions are dominating the bulk chondrite depletion patterns. Higher abundances of volatile elements in matrix relative to bulk suggests recondensation in the matrix of elements lost from chondrules during melting (model a). However, higher matrix enrichments could result from just preferential loss from chondrules without recondensation (model b).

Comparing matrix and bulk, deviations are less for the lithophile (silicate) elements (Ni, Cr, Mn, Lo, K, Rb, Cs in *gray bars* in Fig. 5) and deviations are greater for the siderophile elements (with affinities to metal with Fe) and chalcophile elements (affinities to sulfur). The depletion pattern suggests chondrules are silicates and matrix holds for metal and sulfides (21). Bland *et. al.* (2005) state, "evaporation/recondensation should affect lithophiles as well

as siderophiles and chalcophiles". Thus, his preferred idea is "physical separation of metal and sulfide from the chondrule melt and re-accretion of this material with matrix to explain chondrule/matrix complementarity in siderophiles and chalcophiles." In this scenario, prior to chondrule forming events there was physical separation of silicates, metal and sulfide. Taylor (1993) says this simply(23), "chondrules are mainly silicate because only silicates had aggregated into millimeter-sized lumps before the melting event; the metal and sulfides remained finely dispersed."

In either model (a or b), complimentary compositions existed prior to chondrite accretion of type I chondrules and matrix. In (model b), type I chondrules became isolated from the gas first at high temperature, and matrix formed from fractionated gas left after removal of Mg-rich high temperature components: the transfer of volatiles from mm-size type I chondrules to 100 μm-size and smaller matrix imposes constraints on the size of the region and conditions for chondrule formation. Specifically, models like the X-wind that expel high temperature materials out of chondrule forming regions do not explain complementarity and do not explain the depletion pattern. (See footnote 20). Incomplete nebular condensation produces matrix with a range of compositions and a monotonic decrease in volatile abundance (the depletion pattern) if the ppdisk creates solids all "in one go" whereby gas dissipates as the gas cools (24; 25). However, such global mechanisms when coupled with chondrule formation do not work to produce the depletion pattern (26).

Complementarity between large solids (Mg-rich type I chondrules) and small solids (Fe-rich matrix with type II chondrule olivine) and the depletion pattern are important aspects of chondrule formation. There is no consensus on the origin of the matrix (6). The depletion pattern is an unsolved mystery of chondrule-matrix-chondrite formation.

Mn is one of the suite of moderately volatile elements that participates in the depletion sequence. Mn is moderately volatile because MnO is characterized by having a condensation temperature between the Mg-rich silicates and FeS. Mn prefers to be incorporated into silicates, namely olivines. Mn is systematically enriched in Fe-bearing olivines, and Fe-bearing olivines are an important constituent of the Fe-rich type IIA chondrules. Type II chondrule fragments are part of the matrix. Thus, the Mn-Fe relation is a prominent tool for investigating key aspects of type II chondrule formation.

References

1 Jones RH, Grossman JN, Rubin AE. 2005 Chemical, Mineralogical and Isotopic Properties of Chondrules: Clues to Their Origin. In *Chondrites and the Protoplanetary Disk* (ed. AN Krot, ERD Scott, B Reipurth), volume 341 of *Astronomical Society of the Pacific Conference Series*, p. 251. (doi:10.11150/jFAKEdoi)

2 Jones RH. 2012 Petrographic constraints on the diversity of chondrule reservoirs in the protoplanetary disk. *Meteoritics and Planetary Science* **47**, 1176–1190. (doi:10.1111/j.1945-5100.2011.01327.x)

3 Wooden DH, Harker DE, Brearley AJ. 2005 Thermal processing and radial mixing of dust: Evidence from comets and primitive chondrites. In *Chondrites and the Protoplanetary Disk* (ed. AN Krot, ERD Scott, B Reipurth), volume 341 of *Astronomical Society of the Pacific Conference Series*, p. 774. (doi:10.11150/jFAKEdoi)

4 Scott ERD. 2007 Chondrites and the protoplanetary disk. *Annual Review of Earth and Planetary Sciences* **35**, 577–620. (doi:10.1146/annurev.earth.35.031306.140100)

5 Prinz M, Weisberg MK, Nehru CE. 1988 Gunlock, a New Type 3 Ordinary Chondrite with a Golfball-Sized Chondrule. *Meteoritics* **23**, 297.

6 Huss GR, Alexander CMO, Palme H, Bland PA, Wasson JT. 2005 Genetic relationships between chondrules, fine-grained rims, and interchondrule matrix. In *Chondrites and the Protoplanetary Disk* (ed. AN Krot, ERD Scott, B Reipurth), volume 341 of *Astronomical Society of the Pacific Conference Series*, p. 701. (doi:10.11150/jFAKEdoi)

7 Fedkin AV, Grossman L. 2016 Effects of dust enrichment on oxygen fugacity of cosmic gases. *Meteoritics and Planetary Science* **51**, 843–850. (doi:10.1111/maps.12627)

8 Fedkin AV, Grossman L, Ciesla FJ, Simon SB. 2012 Mineralogical and isotopic constraints on chondrule formation from shock wave thermal histories. Geochim. Cosmochim. Acta **87**, 81–116. (doi:10.1016/j.gca.2012.03.020)

9 Tissandier L, Libourel G, Robert F. 2002 Gas-melt interactions and their bearing on chondrule formation. *Meteoritics and Planetary Science* **37**, 1377–1389. (doi:10.1111/j.1945-5100.2002.tb01035.x)

10 Friend P, Hezel DC, Mucerschi D. 2016 The conditions of chondrule formation, Part II: Open system. Geochim. Cosmochim. Acta **173**, 198–209. (doi:10.1016/j.gca.2015.10.026)

11 Berlin J, Jones RH, Brearley AJ. 2011 Fe-Mn systematics of type IIA chondrules in unequilibrated CO, CR, and ordinary chondrites. *Meteoritics and Planetary Science* **46**, 513–533. (doi:10.1111/j.1945-5100.2011.01171.x)

12 Schrader DL, Connolly HC, Lauretta DS, Zega TJ, Davidson J, Domanik KJ. 2015 The formation and alteration of the Renazzo-like carbonaceous chondrites III: Toward understanding the genesis of ferromagnesian chondrules. *Meteoritics and Planetary Science* **50**, 15–50. (doi:10.1111/maps.12402)

13 Gainsforth Z, Butterworth AL, Stodolna J, Westphal AJ, Huss GR, Nagashima K, Ogliore R, Brownlee DE, Joswiak D, Tyliszczak T, Simionovici AS. 2015 Constraints on the formation environment of two chondrule-like igneous particles from comet 81P/Wild 2. *Meteoritics and Planetary Science* **50**, 976–1004. (doi:10.1111/maps.12445)

14 Schrader DL, Connolly HC, Lauretta DS, Nagashima K, Huss GR, Davidson J, Domanik KJ. 2013 The formation and alteration of the Renazzo-like carbonaceous chondrites II: Linking O-isotope composition and oxidation state of chondrule olivine. Geochim. Cosmochim. Acta **101**, 302–327. (doi:10.1016/j.gca.2012.09.045)

15 Palme H, Hezel DC, Ebel DS. 2015 The origin of chondrules: Constraints from matrix composition and matrix-chondrule complementarity. *Earth and Planetary Science Letters* **411**, 11–19. (doi:10.1016/j.epsl.2014.11.033)

16 Hezel DC, Palme H. 2010 The chemical relationship between chondrules and matrix and the chondrule matrix complementarity. *Earth and Planetary Science Letters* **294**, 85–93. (doi:10.1016/j.epsl.2010.03.008)

17 Jarosewich E. 1990 Chemical analyses of meteorites - A compilation of stony and iron meteorite analyses. *Meteoritics* **25**, 323–337. (doi:10.11150/jFAKEdoi)

18 Stracke A, Palme H, Gellissen M, Münker C, Kleine T, Birbaum K, Günther D, Bourdon B, Zipfel J. 2012 Refractory element fractionation in the Allende meteorite: Implications for solar nebula condensation and the chondritic composition of planetary bodies. Geochim. Cosmochim. Acta **85**, 114–141. (doi:10.1016/j.gca.2012.02.006)

19 Palme H, Larimer JW, Lipschutz ME. 1988 Moderately volatile elements. In *Meteorites and the Early Solar System* (ed. JF Kerridge, MS Matthews), pp. 436–461. University of Arizona Press.

20 Lauretta DS, Nagahara H, Alexander CMO. 2006 Petrology and Origin of Ferromagnesian Silicate Chondrules. In *Meteorites and the Early Solar System II* (ed. DS Lauretta, HY McSween), pp. 431–459. University of Arizona Press.

21 Bland PA, Alard O, Benedix GK, Kearsley AT, Menzies ON, Watt LE, Rogers NW. 2005 Volatile fractionation in the early solar system and chondrule/matrix complementarity. *Proceedings of the National Academy of Science* **102**, 13755–13760. (doi:10.1073/pnas.0501885102)

22 Hewins RH, Zanda B. 2012 Chondrules: Precursors and interactions with the nebular gas. *Meteoritics and Planetary Science* **47**, 1120–1138. (doi:10.1111/j.1945-5100.2012.01376.x)

23 Taylor SR. 1993 *Solar System Evolution, A New Perspective*. Cambridge University Press.

24 Cassen P. 1996 Models for the fractionation of moderately volatile elements in the solar nebula. *Meteoritics and Planetary Science* **31**, 793–806. (doi:10.1111/j.1945-5100.1996.tb02114.x)

25 Cassen P. 2001 Nebular thermal evolution and the properties of primitive planetary materials. *Meteoritics and Planetary Science* **36**, 671–700. (doi:10.1111/j.1945-5100.2001.tb01908.x)

26 Ciesla FJ. 2007 Cooling off the solar nebula: The origin of moderately volatile element depletions in chondritic meteorites. In *Lunar and Planetary Science Conference*, volume 38 of *Lunar and Planetary Science Conference*, p. 1387. (doi:10.11150/jFAKEdoi)
